# Enlarged dendritic spines and pronounced neophobia in mice lacking the PSD protein RICH2

**DOI:** 10.1186/s13041-016-0206-6

**Published:** 2016-03-11

**Authors:** Tasnuva Sarowar, Stefanie Grabrucker, Karl Föhr, Katharina Mangus, Matti Eckert, Juergen Bockmann, Tobias M. Boeckers, Andreas M. Grabrucker

**Affiliations:** WG Molecular Analysis of Synaptopathies, Neurology Department, Ulm University, Albert-Einstein-Allee 11, D-89081 Ulm, Germany; Institute for Anatomy and Cell Biology, Ulm University, Albert-Einstein-Allee 11, D-89081 Ulm, Germany; Department of Anesthesiology, University of Ulm, 89081 Ulm, Germany

**Keywords:** Shank3, Small GTPase, Rac1, Dendritic spine, Autism, Neophobia, Spine morphogenesis, Phobia

## Abstract

**Background:**

The majority of neurons within the central nervous system receive their excitatory inputs via small, actin-rich protrusions called dendritic spines. Spines can undergo rapid morphological alterations according to synaptic activity. This mechanism is implicated in learning and memory formation as it is ultimately altering the number and distribution of receptors and proteins at the post-synaptic membrane, thereby regulating synaptic input. The Rho-family GTPases play an important role in regulating this spine plasticity by the interaction with cytoskeletal components and several signaling pathways within the spine compartment. Rho-GAP interacting CIP4 homologue2/RICH2 is a Rho-GAP protein regulating small GTPases and was identified as an interaction partner of the scaffolding protein SHANK3 at post-synaptic densities.

**Results:**

Here, we characterize the loss of RICH2 in a novel mouse model. Our results show that RICH2 KO animals display a selective and highly significant fear of novel objects and increased stereotypic behavior as well as impairment of motor learning. We found an increase in multiple spine synapses in the hippocampus and cerebellum along with alterations in receptor composition and actin polymerization. Furthermore, we observed that the loss of RICH2 leads to a disinhibition of synaptic RAC1 in vivo.

**Conclusions:**

The results are in line with the reported role of RAC1 activity being essential for activity-dependent spine enlargement. Since SHANK3 mutations are known to be causative for neuropsychiatric diseases of the Autism Spectrum (ASD), a disintegrated SHANK3/RICH2 complex at synaptic sites might at least in part be responsible for abnormal spine formation and plasticity in ASDs.

**Electronic supplementary material:**

The online version of this article (doi:10.1186/s13041-016-0206-6) contains supplementary material, which is available to authorized users.

## Background

Dendritic spines are the sites harboring the post-synaptic compartment of excitatory synapses. These glutamatergic synapses in the central nervous system (CNS) are characterized by an electron dense structure underneath the postsynaptic membrane – the postsynaptic density (PSD). This highly dynamic protein network receives and integrates neurotransmitter signals. The PSD is composed of cell adhesion molecules, membrane bound receptors and channels, G-proteins, scaffolding proteins, cytoskeletal proteins and a wide range of different signaling modulators and effectors [1, 2]. The remodeling of the actin cytoskeleton within a spine is, inter alia, regulated by small GTPases signaling cascades. GTPases act as molecular switches and are active in their GTP-bound form and inactive when bound to GDP. All GTPases of the Rho family (CDC42 (cell division cycle 42), RAC1 (Ras -related C3 botulinum toxin substrates 1), and RhoA (Ras homologous member)) are active in dendritic spines [3, 4] and several studies have shown that growth and stability of synapses is inhibited by RhoA and promoted by RAC1 and CDC42 [4–7]. In this respect, GTPase-modulating proteins play an important role in the postsynaptic compartment by orchestrating a distinct set of G-proteins. The correct interplay of GTPase activating proteins (GAPs) and guanine nucleotide exchange factors (GEFs) is necessary for adequate modulation of the post-synaptic signaling machinery as a reaction to input-signals [8]. GTPases are activated by specific GEFs, the inactivation of the GTP-bound GTPases in turn is controlled by GAPs, which catalyze GTP hydrolysis [4, 5, 9, 10].

RICH2 (RhoSAP: RhoGAP synapse associated protein), which harbors a RhoGAP domain, is highly enriched in the PSD of excitatory synapses. RICH2 is a member of a protein family that also comprises RICH1/Nadrin, and the Abl-binding protein 3BP-1 [9, 11]. RICH2 was identified as an interacting partner of SHANK3 via the PDZ domain [12]. SHANK family members coordinate structural and functional changes within the post-synapse [13] through their direct and indirect interaction with various PSD proteins such as postsynaptic glutamate receptors and the actin cytoskeleton [14–20]. The interaction with SHANK3 might hint towards a participation of RICH2 in synaptic pathways associated with neuropsychiatric diseases since PSD scaffolding proteins of the SHANK family have been closely associated with Autism Spectrum Disorders (ASD) and Schizophrenia (SCZ) [21–23].

To elucidate the role of RICH2 in vivo, we generated mice lacking all isoforms of RICH2. Subsequently, we performed a detailed assessment especially analyzing structural and behavioral parameters. We found a sustained RAC1 activity in RICH2 KO animals that was accompanied with an increased number of PSDs in neurons of the hippocampus and cerebellum. Moreover, mice showed an unusual and highly pronounced novel object anxiety.

## Results

### Generation and characterization of RICH2 mutant mice

**Fig. 1:**
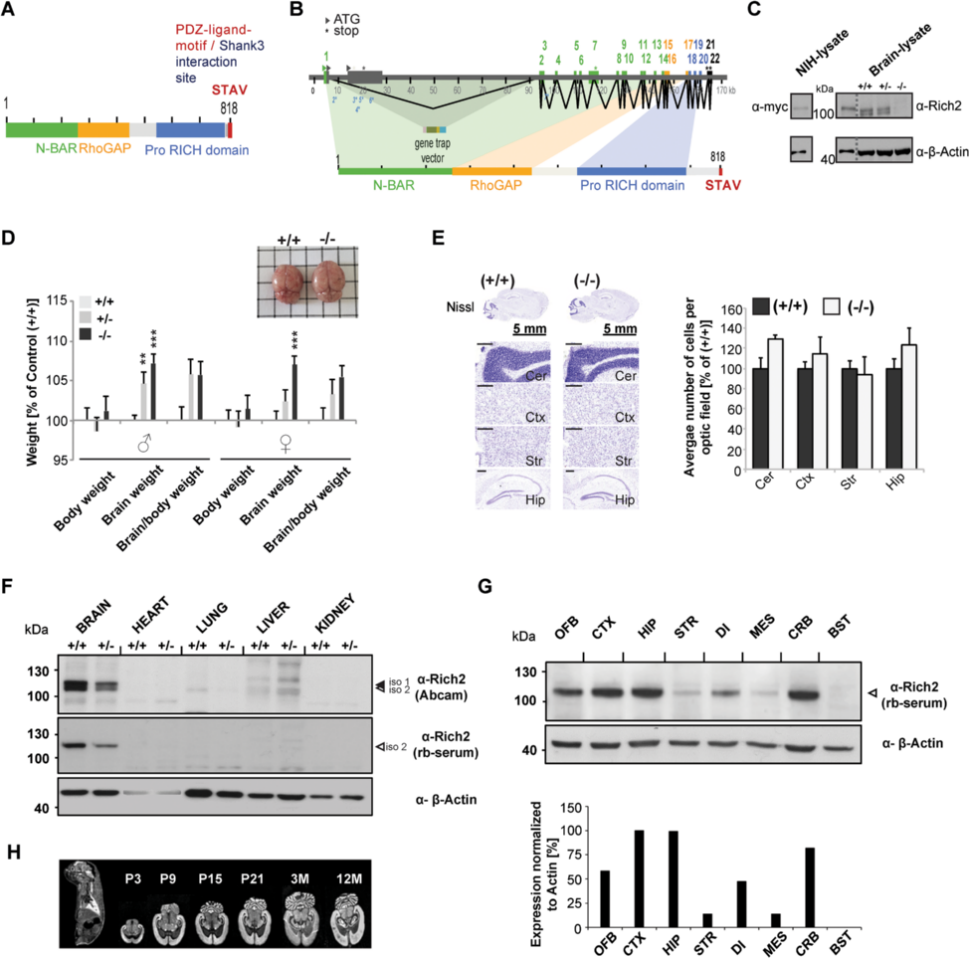
Generation and characterization of RICH2 mutant mice. **a** Illustration of *Rich2* gene (Arhgap44 Rho GTPase activating protein 44 [*Mus musculus*]; NCBI Gene ID: 216831). **b** Domain-coding exons are numbered and colored as the corresponding domain. Sites for initiation of transcription are marked by triangles and stop codons are marked by asterisks. Insertion of the gene trap vector in exon 1 is shown. **c** Western blot illustrating knock-out of the two brain specific RICH2 isoforms in whole brain lysate of RICH2 mutant mice. A gene-dosage effect by knock-out of a single copy of RICH2 in heterozygous mice can be seen by a decreased immunoreactive signal in comparison to the whole brain lysate of wild type mice. To confirm specificity of RICH2 bands, recombinant myc-RICH2, overexpressed in NIH-cells was detected by myc- as well as RICH2-antibody at similar molecular weight. **d** Measuring wet brain weight revealed an increase of approx. 7 % for both genders, whereas body weight remained unchanged (n(♂) = 21 (+/+), 13 (+/−), 13 (−/−); n(♀) = 18 (+/+), 11 (+/−), 12 (−/−)) (One-way ANOVA: (Brain weight male: F_2.44_ = 12.512, *p* = 0.000049, Bonferroni post-hoc analysis: RICH2^+/+^ vs. RICH2^+/−^ *p* < 0.01, RICH2^+/+^ vs RICH2^−/−^ *p* < 0.0001, RICH2^+/−^ vs. RICH2^−/−^ *p* = 0.357; female: F_2.38_ = 7.9793, *p* = 0.0012, post-hoc analysis RICH2^+/+^ vs. RICH2^+/−^ *p* = 0.585, RICH2^+/+^ vs. RICH2^−/−^ *p* < 0.0001, RICH2^+/−^ vs. RICH2^−/−^ *p* = 0.072), (Body weight male: F_2.44_ = 0.47517, *p* = 0.62493; female: F_2.38_ = 0.41256, *p* = 066488)). **e** Quantitative analysis of Nissl staining from three different animals and three different brain sections of similar planes from each group reveals no significant changes between wild type and knock-out mice. Nissl positive signals of three optic fields of view from each brain region and brain section were counted (scale bar = 300 μm) (unpaired *t*-test, Cerebellum: df = 2, *p* = 0.32, *t* = 4.30; Cortex: df = 2, *p* = 0.421, *t* = 4.3026; Striatum: *df* = 2, *p* = 0.4574, *t* = 4.3026; Hippocampus: *df* = 2, *p* = 0.8311, *t* = 4.3026). **f** Western blot of different tissue lysates from P70 wild type and heterozygous mice (brain, heart, lung, liver and kidney) demonstrate RICH2 to be predominantly present in brain lysates and less so in liver. **g** Western blot showing RICH2 expression profile in crude protein lysates of several brain - regions. Cortical, cerebellar and hippocampal lysates show most intense RICH2 immunoreactivities (OFB *olfactory bulb*, CTX *cortex*, HIP *hippocampus*, STR *striatum*, DI *diencephalon*, MES *mesencephalon*, CRB *cerebellum*, BST *brain stem*). **h** *In situ* hybridization also reveals expression of RICH2 mRNA mostly in cortex, cerebellum and hippocampus

To generate RICH2 mutant mice, we used an embryonic cell line (RRZ340) in the vector pGT2Lxf that was genetically modified by random gene-trap insertions from Baygenomics. The selected cell line harbors the gene-trap vector inside the RICH2-gene location (PCR primer sequence for gene trap vector CAG TTT AAC CGC ATG CGC CAG TTG GCC AAC CAG ACG GTG GG) (Fig. 1a, b). The chimeric animals were breeded with C57BL/6 J for ten generations. Finally, breedings between heterozygous animals were carried out in order to generate knock-out mice. A RICH2 specific genomic knock-out can be seen by Western blot analysis of total brain lysate from wild type (**+/+**), heterozygous (**+/−**) and RICH2 knock-out mice (−/−) (Fig. 1c). As size control, myc-RICH2 from RICH2 overexpressing NIH-cells was used. Recombinant myc-RICH2 could be detected at the same molecular weight as the RICH2 protein detected in brain lysates.

Using distinct subcellular brain fractions (CCH, S1, P1, S2, P2), the absence of RICH2 immunoreactivity in fractions from knock-out animals was verified using two different RICH2 antibodies (Additional file 1: Fig. S1a). Given that an additional immunoreactive band at 110 kDa was detected by one antibody (Additional file 1: Fig. S1a, upper series) that also disappeared in knock-out animals, we performed another Western blot series using cortical P2-fractions from wild type and knock-out mice to elucidate the functionality of the available RICH2-antibodies at varying dilutions. As seen in the Western blots on whole brain subcellular fractions, only one prominent band at 120 kDa was detected by all five individual RICH2 antibodies using cortical P2-fractions (two commercial and three serum antibodies) and was not seen in corresponding knock-out lysates (Additional file 1: Fig. S1b).

Using RT-PCR experiments, we verified the location of insertion of the gene trap vector between exon 1 and exon 7 of the RICH2-gene (ARHGAP44). In addition, a chromosomal deletion could be detected covering exon 2 to exon 6 (Additional file 1: Fig. S1c). Another RT-PCR experiment using two sense primers within the gene-trap sequence (GGT GAT GAC GGT GAA AAC CT and CGG TGA AAA CCT CTG ACA CA) and two antisense primers within exon 7 (TGA GCG AAG ACC TTC TCC AG and TGG AGA AGG TCT TCG CTC AG) demonstrated the 3’- end of the gene-trap insertion to be located just next to the 5’- end of exon 7.

Measuring body and brain weight of male as well as female mice at P70 revealed significant changes in wet brain weight (Fig. 1d) (one way ANOVA, male: F_2.44_ = 12.512, *p* = 0.000049, Bonferroni post-hoc analysis: RICH2^+/+^ vs. RICH2^+/−^*p* < 0.01, RICH2^+/+^ vs. RICH2^−/−^*p* < 0.0001, RICH2^+/−^ vs. RICH2^−/−^*p* = 0.357; female: F_2.38_ = 7.9793, *p* = 0.0012, Bonferroni post-hoc analysis RICH2^+/+^ vs. RICH2^+/−^*p* = 0.585, RICH2^+/+^ vs. RICH2^−/−^*p* < 0.0001, RICH2^+/−^ vs. RICH2^−/−^*p* = 0.072), but total body weight remained unchanged in all genotypes (one way ANOVA, male: F_2.44_ = 0.47517, *p* = 0.62493; female: F_2.38_ = 0.41256, *p* = 066488). The increase of brain weight in RICH2^−/−^ mice was seen as clear trend after normalizing individual brain weights to the total body weight of the corresponding mouse (one way ANOVA, male: F_2.44_ = 3.4711, *p* = 0.03156, Bonferroni post-hoc analysis: RICH2^+/+^ vs. RICH2^+/−^*p* = 0.081, RICH2^+/+^ vs. RICH2^−/−^*p* = 0.086, RICH2^+/−^ vs. RICH2^−/−^*p* = 1.000; female: F_2.38_ = 3.13, *p* = 0.055). These differences are not reflected by an increase in cell number per analyzed brain region, although a trend towards an increase across all brain regions in knock-out animals can be seen (unpaired *t*-test, Cerebellum: df = 2, *p* = 0.32, t = 4.30; Cortex: df = 2, *p* = 0.421, t = 4.3026; Striatum: df = 2, *p* = 0.4574, t = 4.3026; Hippocampus: df = 2, *p* = 0.8311, t = 4.3026) (Fig. 1e).

Offspring from breeding of heterozygous mice did not deviate from the expected Mendelian distribution (0.25:0.50:0.25) (Additional file 1: Fig. S1d) and knock-out males and females were able to breed.

Furthermore, we found RICH2 predominately expressed in the brain. The analysis of distinct tissue samples (brain, heart, lung, liver, kidney) by Western blot, detected the mentioned 110 and 120 kDa bands to be present almost exclusively in whole brain lysates (Fig. 1f). Within the brain, the analysis of lysates of several brain regions by Western blot as well as *in situ* hybridization experiments indicate high RICH2 expression in cortex, cerebellum and hippocampus and a moderate to low expression in the olfactory bulb as well as diencephalic subregions (Fig. 1g and h).

### Larger dendritic spines and altered glutamatergic synapses in RICH2^−/−^mice

The RICH2 protein is comprised of a N-terminal BAR (Bin–Amphiphysin–Rvs) domain, a Rho-GAP (Rho GTPase activating protein) domain, a Proline rich domain and a C-terminal PDZ binding (STAV-) motif (Fig. 1a). RICH2 was shown to localize to PSDs of glutamatergic synapses via its interaction with the PDZ domain of SHANK3 [12] (Additional file 2: Fig. S2a and b). Additionally, an effect of RICH2 on AMPAR-recycling and spine morphology was shown after RICH2 overexpression in cultured hippocampal neurons [12, 24].

**Fig. 2:**
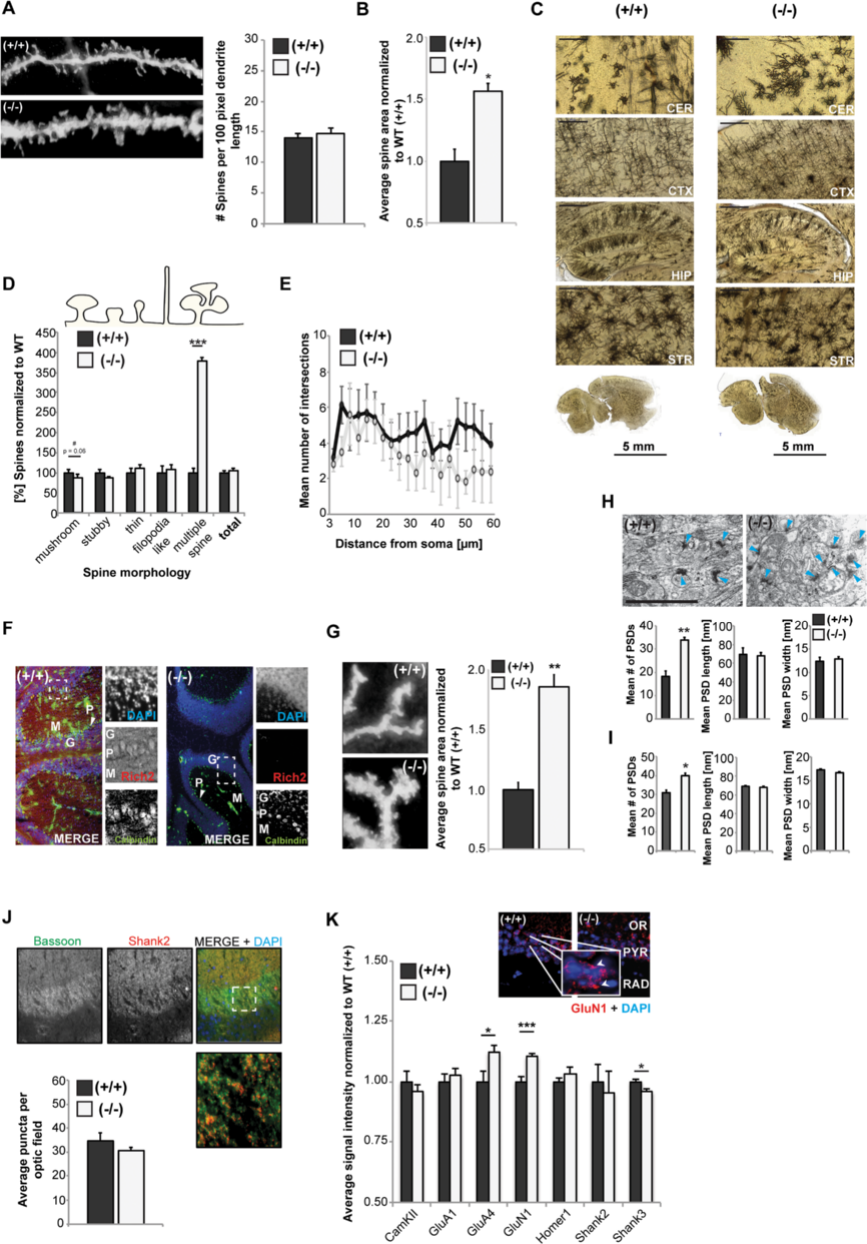
Altered spine morphology and synapse composition in RICH2 mice. **a**-**e** Multiple sections from three wild type and knock-out mice each were subjected to Golgi staining (Cerebellum: CER, Cortex: CTX, Hippocampus: HIP, Striatum: STR) (scale bar = 100 μm (CER) and 200 μm CTX, HIP, STR). **a**–**c** Analyzing the CA1 region from hippocampal sections reveals no significant changes in spine density between wild type and RICH2^−/−^ mice (**a**) (unpaired *t*-test, df = 4, *p* = 0.68, t =3.18). The spine volume is significantly increased in RICH2^−/−^ mice (**b**) (unpaired *t*-test, df = 4, *p* = 0.0121, *t* = 3.8407). **d** Spines were categorized in immature (thin and filopodia like) and mature spines (mushroom, stubby, and multiple spine post-synapses). While no alterations among immature spine types were seen, a shift from mushroom and stubby towards multiple - spine synapses in RICH2^−/−^ mice is visible (1400 (RICH2^+/+^) and 1459 (RICH2^−/−^) spines were calculated from at least 10 cells from 3 animals per genotype) (two way factorial ANOVA, genotype F_1.44_ = 3.013, *p* = 0.095; morphology F_1.44_ = 88.471, *p* < 0.0001, morphology x genotype F_1.44_ = 7.744, *p* < 0.0001; RICH2^+/+^ vs. RICH2^−/−^: Mushroom: *p* = 0.0526; Stubby: *p* = 0.0937; Multiple spine: *p* < 0.0001; Thin: *p* = 0.8038; Filopodia like: *p* = 0.5825) **e**) Using Scholl-analysis after Golgi staining of hippocampal neurons (10 cells per animal, 3 different animals per group), no significant difference in dendritic arborization between knock-out and wild type mice can be detected (repeated measure ANOVA, genotype F_1.114_ = 8.398, *p* = 0.012; distance from soma F_1.114_ = 1.637, *p* = 0.022, distance from soma x genotype F_1.114_ = 1.390, *p* = 0.089). **f** Immunohistochemistry of cerebellar sections of wild type (left panel) and Rich2^−/−^ (right panel) mice shows expression of RICH2 among all cell layers (Molecular layer (M), Purkinje layer (P), Granular layer (G)) with increased labeling of Purkinje cells marked by Calbindin immunoreactivity. **g** Analysis of cerebellar sections using Golgi staining similarly reveals a significantly increased spine volume in RICH2^−/−^ mice (unpaired *t*-test, df = 4, *p* = 0.0013, *t* = 8.0461). **h**, **i** Ultra-structural analysis using transmission electron microscopy of brain sections from RICH2^+/+^ and RICH2^−/−^ cerebellum (**h**) and hippocampus (**i**). **h**) As visible on exemplary images (upper panel, scale bar = 2 μm), the average number of PSDs per optic field of view is significantly increased in RICH2^−/−^ mice (unpaired *t*-test, Cerebellum: df = 4, *p* = 0.0044, *t* = 5.851; Hippocampus: df = 4, *p* = 0.0106, *t* = 4.5317). No change in average PSD length and width was observed in cerebellum (analysis was performed using 3 animals per genotype, 12 optic field of view per animal with a total of 581RICH2^+/+^ and 754 RICH2^−/−^ PSDs analyzed) and in the hippocampal CA1 region (analysis was performed using 3 animals per genotype, 12 optic field of view per animal with a total of 657 RICH2^+/+^ and 1154 RICH2^−/−^ PSDs analyzed) (unpaired *t*-test, Cerebellum: length df = 4, *p* = 0.8622, *t* = 0.1850; width df = 4, *p* = 0.6441, *t* = 0.4989; Hippocampus: length df = 4, *p* = 0.6298, *t* = 0.5211; width df = 4, *p* = 0.3213, *t* = 1.1309). **j** Immunohistochemistry, similar to Golgi staining, did not reveal significant changes in synapse density between wild type and RICH2^−/−^ mice in stratum pyramidale (PYR), stratum oriens (OR), and stratum radiatum (RAD) of dendritic spines from hippocampal CA1 pyramidal neurons using unfixed fresh frozen brain sections (14 μm) from wild type and RICH2^−/−^ animals (*n* = 4 / genotype, 2 analyzed section per animal). Sections were analyzed regarding the number of immunoreactive puncta per optic field (unpaired *t*-test, df = 4, *p* = 1.000, *t* = 2.7764). **k** Histological immunostainings of CamKIIα/β, GluA1, GluA4, GluN1, Homer1, SHANK2 and SHANK3 in stratum pyramidale (PYR), stratum oriens (OR), and stratum radiatum (RAD) of dendritic spines from hippocampal CA1 pyramidal neurons. Sections were analyzed regarding the average signal intensity per immunoreactive puncta per optic field. A significant increase for GluA4 and GluN1 can be seen in knock-out animals (unpaired *t*-test, GluA4: df = 18, *p* = 0.0294, *t* = 2.3659; GluN1: df = 18, *p* = 0.0003, *t* = 4.4109), while the signal intensity and thus protein levels for SHANK3 show a significant decrease in knock-out mice (df = 18, *p* = 0.0216, *t* = 2.5151)

Thus, having confirmed the successful knock-out of RICH2, we next investigated the effects of RICH2 knock-out on synaptic spines. We performed Golgi staining of 3 brains each from wild type and RICH2^−/−^mice at P70 (Fig. 2a-e and g). RICH2^−/−^ mice do not show a significant change in spine density (Fig. 2a) analyzed in the hippocampal CA1 region (unpaired *t*-test, df = 4, *p* = 0.68, t = 3.18). However, the spine volume was significantly increased (df = 4,* p* = 0.0121, t =3.8407) in the hippocampal CA1 region of RICH2^−/−^ mice (Fig. 2b).

We next categorized the spines based on their morphology and found that there was a significant alteration between the genotypes (two way factorial ANOVA, genotype F_1.44_ = 3.013, *p* = 0.095; morphology F_1.44_ = 88.471, *p* < 0.0001, morphology x genotype F_1.44_ = 7.744, *p* < 0.0001). A shift from mushroom and stubby towards multiple spine synapses was observed (unpaired *t*-test, Mushroom: df = 4, *p* = 0.0526, t = 2.7278, Stubby df = 4, *p* = 0.0937, t = 2.0659; Multiple spine: df = 4, *p* < 0.0001, t = 13.8779). No alterations among immature spine types (thin and filopodia like) were seen (Thin: df = 4, *p* = 0.8038, t = 0.2620; Filopodia like: df = 4, *p* = 0.5825, t = 0.5873) (Fig. 2d). The dendritic arborization of CA1 hippocampal neurons was not altered (repeated measure ANOVA, genotype F_1.114_ = 8.398, *p* = 0.012, distance from soma F_1.114_ = 1.637, *p* = 0.022, distance from soma x genotype F_1.114_ = 1.390, *p* = 0.089) (Fig. 2e). In cerebellum, RICH2 is expressed in all neuronal cell types with slightly higher expression in Purkinje cells (Fig. 2f). An increase in spine volume can also be seen in cerebellar neurons (unpaired *t*-test, df = 4, *p* = 0.0013, t = 8.0461) (Fig. 2g). On ultra-structural level, the shift towards an increased number of multiple spine synapses was confirmed. TEM analyses show a significant increase in the number of PSDs in cerebellum (Fig. 2h) and hippocampus (Fig. 2i) of RICH2^−/−^ animals compared to wild type (unpaired *t*-test, Cerebellum: df = 4, *p* = 0.0044, *t* = 5.851; Hippocampus: df = 4, *p* = 0.0106, *t* = 4.5317). The length and width of PSDs was not significantly altered (Cerebellum: length df = 4, *p* = 0.8622, t = 0.1850; width df = 4, *p* = 0.6441, *t* = 0.4989; Hippocampus: length df = 4, *p* = 0.6298, *t* = 0.5211; width df = 4, *p* = 0.3213, *t* = 1.1309).

Using immunohistochemical read-outs that are able to detect spines but not single PSDs on spines, no significant change in spine density between wild type and RICH2^−/−^ mice in hippocampal CA1 pyramidal neurons was detected. Spines were identified by co-localized labeling of a pre-synaptic maker protein (Bassoon) and a post-synaptic marker (SHANK2) (unpaired *t*-test, *df* = 4, *p* = 1.000, *t* = 2.7764) (Fig. 2j). The post-synaptic marker was chosen based on the results obtained through the analysis of the average signal intensity per immunoreactive puncta per optic field, where no changes in SHANK2 levels could be detected (unpaired *t*-test, *df* = 18, *p* = 0.7062, *t* = 0.4051) (Fig. 2k). However, a significant increase for GluA4 and GluN1 can be seen in knock-out animals (GluA4: df = 18, *p* = 0.0294, t = 2.3659; GluN1: df = 18, *p* = 0.0003, t = 4.4109), while the signal intensity, and thus protein level, for SHANK3 shows a significant decrease in knock-out mice (unpaired *t*-test, df = 18, *p* = 0.0216, *t* = 2.5151) (Fig. 2k).

### Altered synaptic protein composition in RICH2^−/−^mice

**Fig. 3:**
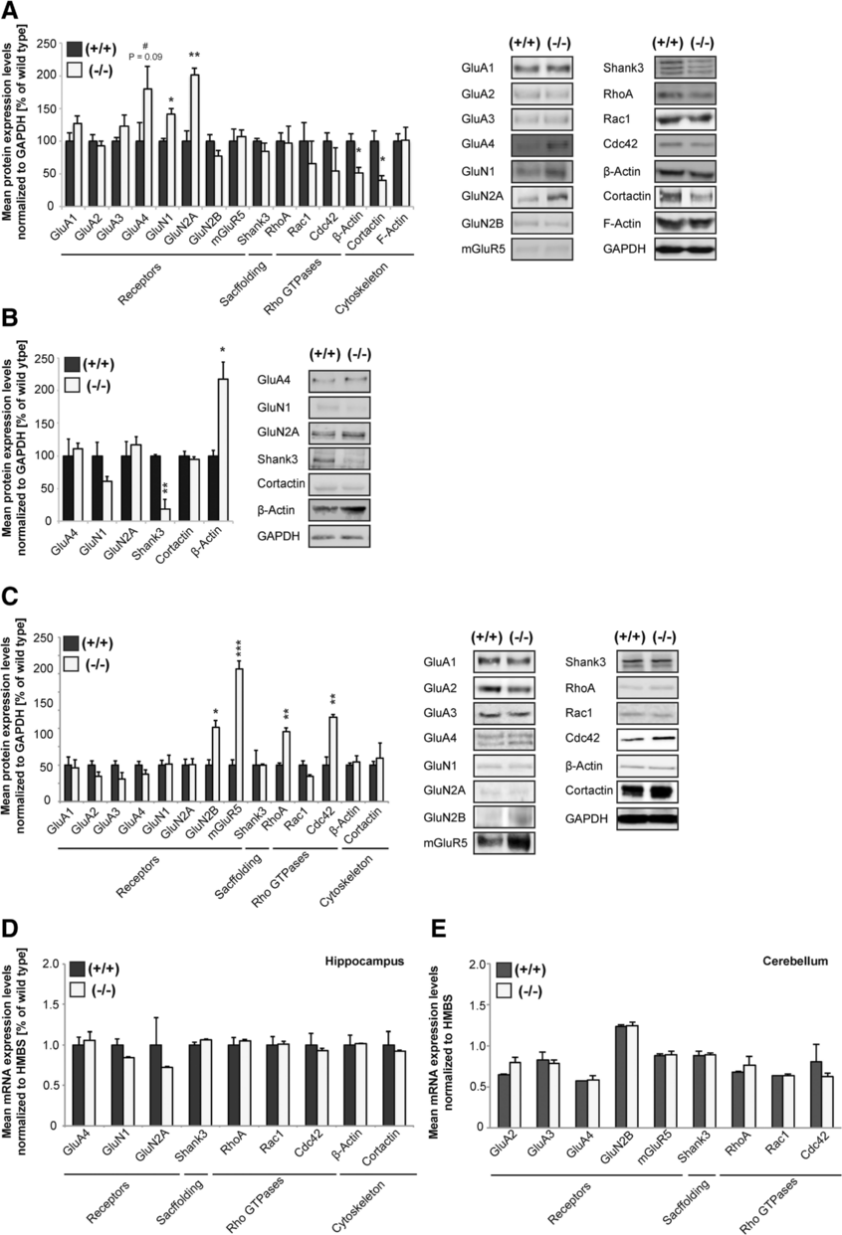
Altered synapse composition and function in RICH2^−/−^ mice. a Western blot analysis (showing relative percentages of mean + SEM) of hippocampal synapse-enriched P2-fractions extracted from P70 wild type (+/+, *n* = 3), and knock-out (−/−, *n* = 3) mice. Proteins were normalized to GAPDH expression levels. Right panel: Representative illustration from hippocampal P2-immunoblots. For each protein analyzed two representative immunonblot-signals are illustrated per genotype. A significant increase can be seen for GluN1 and GluN2A levels, while a significant decrease is visible for β-Actin and Cortactin (unpaired *t*-test, GluN1: df = 4, *p* = 0.0102, *t* = 4.4439; GluN2A: df = 4, *p* = 0.005, *t* = 5.5458; β – Actin: df = 4, *p* = 0.0259, *t* = 3.463; Cortactin: df = 4, *p* = 0.0264, *t* = 3.3999). **b** Western blot analysis (showing relative percentages of mean + SEM) of hippocampal S2-fractions extracted from P70 wild type (+/+, *n* = 3), and knock-out (−/−, *n* = 3) mice. Proteins were normalized to GAPDH expression levels. Right panel: Representative illustration from hippocampal S2-immunoblots. For each protein analyzed two representative immunonblot-signals are illustrated per genotype. **c** Western blot analysis (showing relative percentages of mean + SEM) of cerebellar synapse-enriched P2-fractions extracted from P70 wild type (+/+, *n* = 3), and knock-out (−/−, n = 3) mice. Proteins were normalized to GAPDH expression levels. Right panel: Representative illustration from cerebellar P2-immunoblots. For each protein analyzed two representative immunonblot-signals are illustrated per genotype. A significant increase in protein expression levels can be seen for GluN2B, mGluR5, RHOA, and CDC42 (unpaired *t*-test, GluN2B: df = 4, *p* = 0.0126, *t* = 4.2997; mGluR5: df = 4, *p* = 0.0006, *t* = 10.0161; RhoA: df = 4, *p* = 0.0011, *t* = 8.4287; CDC42: df = 4, *p* = 0.005, *t* = 5.5827). Note that expressions of GluN2B and mGluR5 in general were found to be very low in cerebellar lysates. **d, e** qRT-PCR analysis showing relative changes in hippocampal and cerebellar mRNA-levels (normalized to HMBS) using total RNA extracted from crude cellular homogenate from P70 wild type (+/+, *n* = 3) and knock-out (−/−, *n* = 3) mice. Each qRT-PCR experiment was set up of 3 biological and 3 technical replicates. The results show no significant differences of tested genes between wild type and RICH2^−/−^ mice in hippocampus (unpaired *t*-test, GluA4: df = 4, *p* = 0.7060, *t* = 0.105956; GluN1: df = 4, *p* = 0.1175, *t* = 1.9897; GluN2A: df = 4, *p* = 0.4582, *t* = 0.8201; Shank3: df = 4, *p* = 0.1658, *t* = 1.6924; Cortactin: df = 4, *p* = 0.6827, *t* = 0.4399; β actin: df = 4, *p* = 0.9026, *t* = 0.1303; RhoA: df = 4, *p* = 0.593, *t* = 0.58; CDC42: df = 4, *p* = 0.6591, *t* = 0.4756; Rac1: df = 4, *p* = 0.931, *t* = 0.0922) (**d**) and cerebellum (GluA2: df = 4, *p* = 0.0836, *t* = 2.2929; GluA3: df = 4, *p* = 0.7140, *t* = 0.3936; GluA4: df = 4, *p* = 0.7553, *t* = 0.3338; GluN2B: df = 4, *p* = 0.8972, *t* = 0.1377; mGluR5: *df* = 4, *p* = 0.8873, *t* = 0.1509; Shank3: df = 4, *p* = 0.9134, *t* = 0.1158; RhoA: df = 4, *p* = 0.4049, *t* = 0.9303; CDC42: df = 4, *p* = 0.4359, *t* = 0.8649; Rac1: df = 4, *p* = 0.9799, *t* = 0.0268) (**e**)

To confirm the findings on altered protein levels of PSD enriched proteins and to extend the analysis, we performed Western blot experiments using hippocampal and cerebellar subcellular synapse-enriched P2 protein fractions of P70 mice. The results showed, similar to the results from IHC analysis, increased concentrations of GluA4 (as trend) and GluN1 and GluN2A (significant) (unpaired *t*-test, GluA4: df = 10, *p* = 0.0994, t = 1.8048; GluN1: df = 4, *p* = 0.0102, t = 4.4439; GluN2A: df = 4, *p* = 0.005, t = 5.5458). β - Actin and cortactin levels in turn were significantly reduced in knock-out mice compared to wild type animals (Fig. 3a) in hippocampal fractions (unpaired *t*-test, β – Actin: df = 4, *p* = 0.0259, t = 3.463; Cortactin: df = 4, *p* = 0.0264, t = 3.3999). The alterations in the P2 fraction were in part accompanied by changes of proteins levels in the S2 fraction (Fig. 3b). For example, the decrease in β - Actin and increase in NR1 in the P2 fraction is reflected by and increase of β - Actin and decrease in NR1 in the S2 fraction hinting towards a shift of the protein between different pools (unpaired *t*-test, β – Actin: df = 4, *p* = 0.0115, t = 4.4251; NR1: df = 4, *p* = 0.1445, t = 1.8102). In contrast, SHANK3 levels also decrease in the S2 fraction leading to an overall reduction of SHANK3 in RICH2^−/−^ mice (unpaired *t*-test, Shank3: df = 4, *p* = 0.0067, t = 5.156). No alterations were detected for GluA4, GluN2A and cortactin in the S2 fraction (Fig. 3b) (GluA4: df = 4, *p* = 0.708, t = 0.4023; GluN2A: df = 4, *p* = 0.5304, t = 0.6861; Cortactin: df = 4, *p* = 0.5004, t = 0.74). Unlike hippocampal P2 fractions, analysis of lysate from cerebellar P2 fractions reveals a significant increase in protein expression for GluN2B, mGluR5, RhoA, and CDC42 (unpaired *t*-test, GluN2B: df = 4, *p* = 0.0126, t = 4.2997; mGluR5: df = 4, *p* = 0.0006, t = 10.0161; RhoA: df = 4, *p* = 0.0011, t = 8.4287; CDC42: df = 4, *p* = 0.005, t = 5.5827). However, expressions of GluN2B and mGluR5 in general were found to be very low in cerebellar lysate, which might obfuscate the analysis (Fig. 3c).

To see whether the alterations occur on translational or transcriptional level, we quantified mRNA concentrations of the several genes that displayed differences on protein level (Fig. 3d and e). Alterations of PSD proteins measured in hippocampal protein lysates could not be found on mRNA level using total RNA extracted from hippocampal crude cellular homogenate (Fig. 3d) (unpaired *t*-test, GluA4: df = 4, *p* = 0.7060, t = 0.105956; GluN1: df = 4, *p* = 0.1175, t = 1.9897; GluN2A: df = 4, *p* = 0.4582, t = 0.8201; Shank3: df = 4, *p* = 0.1658, t = 1.6924; Cortactin: df = 4, *p* = 0.6827, t = 0.4399; β actin: df = 4, *p* = 0.9026, t = 0.1303; RhoA: df = 4, *p* = 0.593, t = 0.58; CDC42: df = 4, *p* = 0.6591, t = 0.4756; Rac1: df = 4, *p* = 0.931, t = 0.0922). Similarly, no alterations in mRNA expression were found in cerebellar lysate (Fig. 3e) (GluA2: df = 4, *p* = 0.0836, t = 2.2929; GluA3: df = 4, *p* = 0.7140, t = 0.3936; GluA4: df = 4, *p* = 0.7553, t = 0.3338; GluN2B: df = 4, *p* = 0.8972, t = 0.1377; mGluR5: df = 4, *p* = 0.8873, t = 0.1509; Shank3: df = 4, *p* = 0.9134, t = 0.1158; RhoA: df = 4, *p* = 0.4049, t = 0.9303; CDC42: df = 4, *p* = 0.4359, t = 0.8649; Rac1: df = 4, *p* = 0.9799, t = 0.0268).

### Altered synaptic signaling in RICH2^−/−^mice

**Fig. 4:**
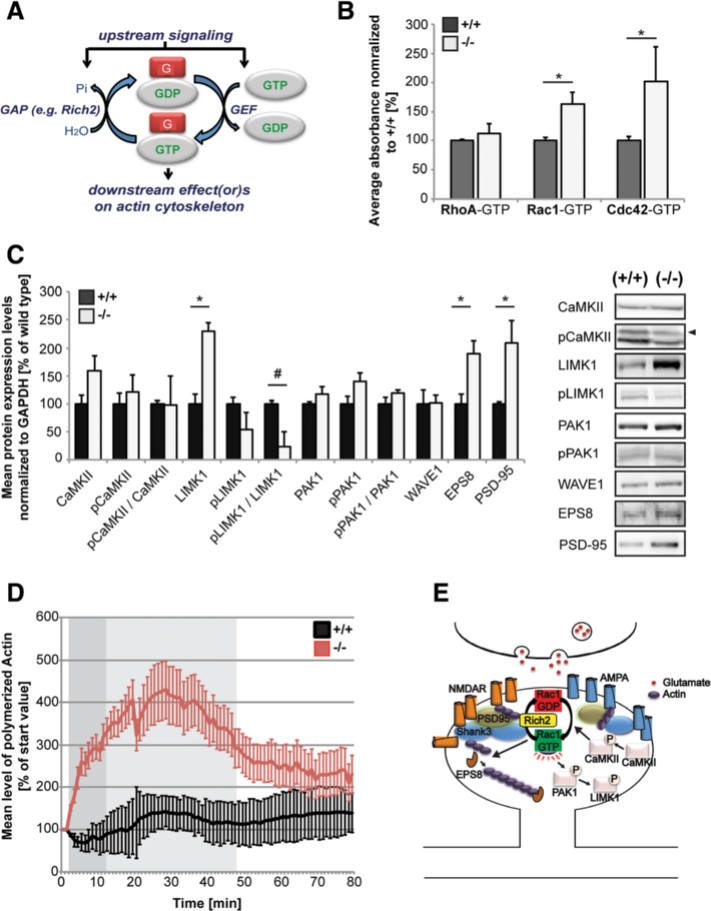
Functional analysis of impaired synaptic signaling caused by deletion of RICH2. **a** Schematic illustration of small G-protein GTPase-cycle. (*G* = G-protein (e.g. *RHO*, *RAC1*, *CDC42)*, *GAP* = *GTPase activating protein*, *GEF* = *guanine-nucleotide exchange factor*, *Pi* = phosphate). G proteins are activated via GEF (guanine nucleotide exchange factor) upon replacement of GDP by GTP and inactivated via GAP (GTPase activating protein) due to hydrolysis of GTP via GAPs. **b** Using G-LISA activation assays, RHOA, RAC1, and CDC42 activity was measured in P2 fractions from hippocampal tissue lysates of three animals per group in technical triplicates. The results show a significantly increased activation (GTP-binding) of RAC1 and CDC42 in RICH2^−/−^ mice compared to wild type controls (unpaired *t*-test, Rac1: df = 4, *p* = 0.0476, *t* = 2.8243; Cdc42: df = 4, *p* = 0.0275, *t* = 3.3908). No significant difference was detected for RhoA (unpaired *t*-test, df = 4, *p* = 0.4727, *t* = 0.7919). **c** Western blot analysis (showing relative percentages of mean + SEM) of hippocampal synapse-enriched P2-fractions extracted from P70 wild type (+/+, *n* = 3), and knock-out (−/−, *n* = 3) mice. Proteins were normalized to GAPDH expression levels. A significant increase in LIMK1 expression levels can be observed in RICH2^−/−^ animals (unpaired *t*-test, LIMK: df = 4, *p* = 0.005, *t* = 5.6029). Given that the levels of phosphorylated LIMK1 (pLIMK1) remain unchanged, a difference in the ratio of pLIMK1 / LIMK1 can be observed, however, only as trend (pLIMK/LIMK: df = 4, *p* = 0.0545, *t* = 2.6923). In addition, the levels of EPS8 and PSD-95 are significantly increased in RICH2^−/−^ mice (unpaired *t*-test, EPS8: *df* = 4, *p* = 0.0384, *t* = 3.039; PSD-95: df = 4, *p* = 0.0529, t = 2.7223). Right panel: Representative illustration from hippocampal P2-immunoblots. For each protein analyzed two representative immunonblot-signals are illustrated per genotype. **d**) Hippocampal P2 lysates from RICH2^+/+^ and RICH2^−/−^ animals (*n* = 3) were used in an actin polymerization assay. The lysate was added to a solution with pyrene-conjugated actin and the increase in fluorescence intensity that occurs when pyrene G-actin (monomer) forms pyrene F-actin measured over a time-course of 80 min. P2 lysate from RICH2^−/−^ mice is able to induce actin polymerization to a significantly higher amount within the first 30 min of the experiment compared to lysate from RICH2^+/+^ mice (repeated measure ANOVA, effect of genotype by time F_1.14_ = 5.402, *p* < 0.0001; effect of time F_1.14_ = 6.397, *p* < 0.0001; effect of the genotype F_1.14_ = 9.015, *p* = 0.040). **e**) Schematic overview of RAC1 downstream signaling pathways. In RICH2^−/−^ mice, effects on spine morphology and actin polymerization most likely are mediated by activation of EPS8

To gain mechanistic insights, we next investigated whether RICH2 knock-out affects the activity of GTPases of the Rho family. We evaluated changes in the ratio between GTP-bound and total GTPase levels (Fig. 4a) comparing P2 wild type brain homogenates from hippocampus to lysates from RICH2^−/−^mice. To that end, we used ELISA-based assays for quantification of CDC42, RAC1, and RhoA GTPases in the GTP-bound state. Using G-LISA activation assays, we detected a significantly increased activation of RAC1 and CDC42 in RICH2^−/−^ mice compared to wild type controls (unpaired *t*-test, Rac1: *df* = 4, *p* = 0.0476, *t* = 2.8243; Cdc42: *df* = 4, *p* = 0.0275, *t* = 3.3908). No significant difference was detected for RHOA (unpaired *t*-test, *df* = 4, *p* = 0.4727, *t* = 0.7919) (Fig. 4b). Given that Western blot analysis of hippocampal P2 tissue homogenates showed no significant increase of total RhoA, RAC1 or CDC42 levels but a trend towards a decrease, the increased amounts of GTP-bound RAC1 and CDC42 were not caused by a total increase in RAC1 and CDC42 protein content, but might in turn even be an underestimation of the activation levels.

Given that specificity in the process of RAC1 activation is thought to arise through control of different downstream targets, we next investigated the signaling cascades triggered by increased activation of RAC1 in RICH2^−/−^ mice in more detail (Fig. 4c-e). We analyzed several known key proteins involved in downstream processes of the RAC1 pathway. For example, RAC1 is able to activate kinases such as PAK1 (p21-activated kinase 1) acting on LIMK1 (LIM domain kinase 1) by increasing the phosphorylation status. However, although we could detect a significant increase in synaptic total LIMK1 protein levels in P2 hippocampal lysates, the ratio of phosphorylated protein to total protein level was unchanged both for PAK1 and LIMK1 (unpaired *t*-test, LIMK: *df* = 4, *p* = 0.005, *t* = 5.6029; pLIMK/LIMK: *df* = 4, *p* = 0.0545, *t* = 2.6923; pPAK1/PAK1: *df* = 4, *p* = 0.2005, *t* = 1.531) (Fig. 4c). Furthermore, via the insulin receptor substrate of 53 kDa (IRSp53), an essential mediator between activated Rac or CDC42, RAC1 acts on effectors such as WAVE (Wiskott–Aldrich syndrome protein (WASP) family verprolin-homologous protein) and EPS8 (Epidermal growth factor receptor kinase substrate 8), which are known regulators of actin dynamics. Indeed, we were able to detect a significant increase in synaptic EPS8 protein levels in RICH2^−/−^ mice (unpaired *t*-test, *df* = 4, *p* = 0.0384, *t* = 3.039) (Fig. 4c). Along with reports showing that an increase in EPS8 leads to an increase in spines containing PSD-95 [25], we detected higher synaptic PSD-95 protein levels in RICH2^−/−^ mice compared to controls (unpaired *t*-test, *df* = 4, *p* = 0.0529, *t* = 2.7223).

To confirm altered actin dynamics, we performed an actin polymerization assay (Fig. 4d) using hippocampal P2 lysate from three wildtype and three RICH2^−/−^ mice. Adding the lysate containing the effectors of actin polymerization, an enhanced fluorescence of pyrene conjugated actin that occurred when pyrene G-actin (monomer) forms pyrene F-actin was measured over time to follow polymerization. The results show indeed that P2 lysate from RICH2^−/−^ mice is able to induce actin polymerization to a significantly higher amount within the first 30 min of the experiment (Fig. 4d) compared to lysate from wildtype mice (repeated measure ANOVA, effect of genotype x time F_1.14_ = 5.402, *p* < 0.0001; effect of time F_1.14_ = 6.397, *p* < 0.0001; effect of the genotype F_1.14_ = 9.015, *p* = 0.040).

Similarly, hippocampal cells from RICH2^−/−^ mice (Additional file 2: Fig. S2c-e) showed a highly significant increase in GTP bound RAC1 assayed by a RAC1-GTP specific ELISA assay (unpaired *t*-test, *df* = 4, *p* = 0.0001, *t* = 16.6757) (Additional file 2: Fig. S2d). Synapse density (data not shown) and dendritic branching (Additional file 2: Fig. S2f) were unchanged (unpaired *t*-test, primary: *df* = 4, *p* = 0.0824, *t* = 2.3062; secondary: *df* = 4, *p* = 0.8492, *t* = 0.2027; tertiary: *df* = 4, *p* = 0.1475, *t* = 1.7925; quaternary: *df* = 4, *p* = 0.96, *t* = 0.0534; total: *df* = 4, *p* = 0.3333, *t* = 1.0995), as was synaptic function *in vitro* using electrophysiological measurements (unpaired *t*-test, frequency: *df* = 30, *p* = 0.4494, *t* = 0.7665; rise time: *df* = 30, *p* = 0.5133, *t* = 0.6615; decay time: *df* = 30, *p* = 0.2637, *t* = 1.1391) (Additional file 2: Fig. S2g), although a tendency towards an increased amplitude of mEPSCs (unpaired *t*-test, *df* = 30, *p* = 0.1733, *t* = 1.3949) and a significantly increased mEPSC signal area were detected in RICH2 depleted cells (*df* = 30, *p* = 0.0492, *t* = 2.05).

However, again, we were able to detect altered synaptic levels of actin. The mean fluorescence intensity of synaptic actin signals co-localizing with Homer1b/c was increased in cells from RICH2^−/−^mice (unpaired *t*-test, Actin: *df* = 18, *p* = 0.0005, *t* = 4.1906; Homer1: *df* = 18, *p* = 0.0103, *t* = 2.8653). Similarly, a trend towards an increase of synaptic actin signal area compared to wild type mice can be seen (unpaired *t*-test, *df* = 18, *p* = 0.0993, *t* = 1.738) (Additional file 2: Fig. S2h and j). This is in line with the data presented above on actin levels and spine enlargement and underlines a role for RICH2-RAC1 in actin polymerization.

### Altered behavior in RICH2^−/−^mice

**Fig. 5:**
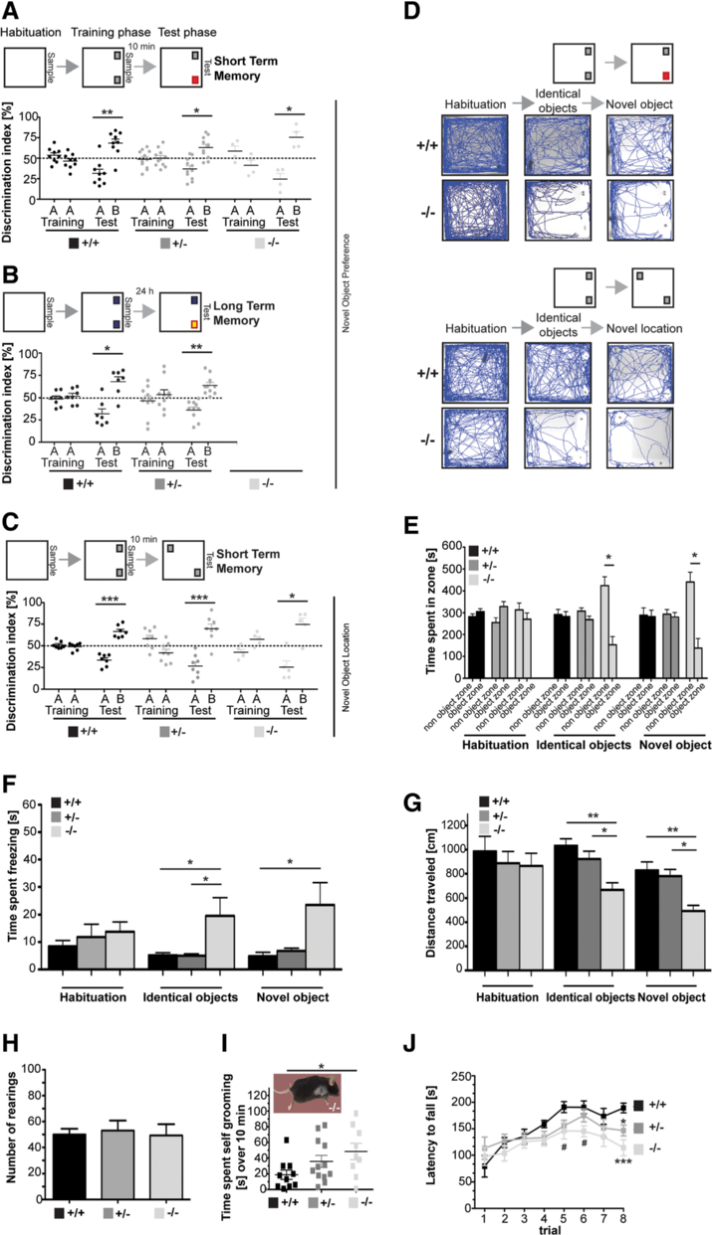
Repetitive behavior, fear of novel objects, and impaired motor learning in RICH2^−/−^ mice. **a**–**d** Novel object recognition task for short and long term memory. **a**) Percent time of mice spent exploring during different stages of the novel object recognition task for short-term memory. Mice were exposed to two identical objects during the training session (AA). 10 min later during the test session, the animals were exposed to a familiar object (A) and a novel object (B). All genotypes showed a significant preference for the novel object after 10 min (two-way mixed ANOVA, genotype by target interaction F_2.21_ = 1.252, *p* = 0.306; target main effect F_1.21_ = 37.531, *p* < 0.001; Post-tests of target effect in all three genotypes: RICH2^+/+^ *p* = 0.006 (*n* = 9); RICH2^+/−^ *p* = 0.007 (*n* = 11); RICH2^−/−^ *p* = 0.034 (*n* = 4)). **b** Percent time of mice spent exploring during different stages of the novel object recognition task for long term memory. Long term memory was assessed 24 h after the training session (10 min exposure to the familiar object (A)). Wild type animals as well as RICH2^+/−^ showed a significant preference for the novel object (B) after 24 h (two-way mixed ANOVA, genotype by target interaction F_1.15_ = 0.412, *p* = 0.531; target main effect F_1.15_ = 28.305, *p* < 0.001; Post-tests of target effect in genotypes: RICH2^+/+^ *p* = 0.016 (*n* = 7); RICH2^+/−^ *p* = 0.003 (*n* = 11)). Analysis of RICH2^−/−^could not be performed since mice which explored objects less than 10 s were excluded from the analysis. Only two RICH2^−/−^ met the criteria for inclusion and thus no conclusion for long term memory can be drawn based on the remaining two RICH2^−/−^ animals for statistical analysis. **c**) Percent time of mice spent exploring during different stages of the novel location task for spatial working memory. Mice were exposed to two identical objects, during the training session. 10 min later, one of the familiar object was moved to a novel location. A significant preference for the “moved” object during the test session for each genotype was measured (two-way mixed ANOVA, genotype by target interaction F_2.17_ = 0.575, *p* = 0.236; target main effect F_1.17_ = 90.745, *p* < 0.001; Post-tests of target effect in genotypes: RICH2^+/+^ *p* = 0.001 (*n* = 7); RICH2^+/−^ *p* = 0.001 (*n* = 8); RICH2^−/−^ *p* = 0.028 (*n* = 5)). **d** Tracking path of mice before and after the presentation of novel objects during the novel object recognition task as well as novel location task. RICH2^−/−^mice actively avoid spending time in the proximity of the novel object and show reduced locomotor response towards novel objects. **e** The open field arena was separated in a non object and object zone during the novel object recognition test. There was a significant difference between genotypes regarding preference for a zone after introduction of objects (two-way mixed ANOVA, genotype and test session interaction F_4.56_ = 2.557, *p* = 0.049; main effect of the genotype F_2.28_ = 10.613, *p* < 0.001; main effect of the test session F_2.56_ = 13.607, *p* = 0.001). No side preference was present in the habituation phase of the novel object recognition test (open field arena without objects) (two-way mixed ANOVA, genotype and zone interaction F_2.28_ = 2.544, *p* = 0.097; main effect of the zone F_1.28_ = 1.044, *p* = 0.316: main effect of the genotype F_2.28_ = 0.718, *p* = 0.497). RICH2^−/−^ mice showed a significant preference for the non object zone in comparison to the object zone in both ‘identical objects test’ (two-way mixed ANOVA, genotype and zone interaction F_2.28_ = 8.608, *p* = 0.001; main effect of the zone F_1.28_ = 16.567, *p* = 0.372: main effect of the genotype F_2.28_ = 0.016, *p* = 0.984) (RICH2^−/−^ *p* = 0.01) and ‘novel object test’ (two-way mixed ANOVA, genotype and zone interaction F_2.28_ = 8.744, *p* = 0.001; main effect of the zone F_1.28_ = 10.795, *p* = 0.003: main effect of the genotype F_2.28_ = 0.131, *p* = 0.878) (RICH2^−/−^ *p* = 0.011). **f** A significant difference among genotypes was detected regarding freezing behavior after introduction of novel objects (two-way mixed ANOVA, genotype by test session interaction F_4.56_ = 3.280, *p* = 0.04; main effect of test session F_2.56_ = 0.478, *p* = 0.623, main effect of the genotype F_2.28_ = 5.452, *p* = 0.01). No significant difference in freezing behavior was found during the habituation phase of the novel object recognition test. However, significant genotype differences were found during identical object presentation (Post-test RICH2^+/+^ vs RICH2^−/−^ *p* = 0.019; RICH2^+/−^ vs RICH2^−/−^ *p* = 0.011). Additionally, during novel object presentation significant differences among genotypes (Post-test RICH2^+/+^ vs RICH2^−/−^ *p* = 0.012; RICH2^+/−^ vs RICH2^−/−^ *p* = 0.017) were detected. **g** Further, a significant genotype difference was found measuring track length (two-way mixed ANOVA, genotype by test session interaction F_4.56_ = 1.739, *p* = 0.194; main effect of test session F_2.56_ = 8.612, *p* = 0.003, main effect of the genotype F_2.28_ = 7.399, *p* = 0.003). No significant difference in track length was detected during the habituation phase of the novel object recognition test. However, significant genotype differences were found during identical object presentation (Post-test RICH2^+/+^ vs RICH2^−/−^ *p* = 0.002, RICH2^+/−^ vs RICH2^−/−^ *p* = 0.023). Additionally, during novel object presentation, a significant difference among genotypes (Post-test RICH2^+/+^ vs RICH2^−/−^ *p* = 0.002, RICH2^+/−^ vs RICH2^−/−^ *p* = 0.01) was detected. **h** Number of rearings in novel environment. No significant difference of RICH2^−/−^ mice compared to wild type animals was detected in general exploratory behavior (F_2.26_ = 0.083, *p* = 0.920; one way ANOVA). **i** For the analysis of ASD-like behavior, repetitive self-grooming was measured over a period of 10 min. RICH2^−/−^ mice display a significant increase in time spent self grooming in comparison to wild type mice. A One Way Analysis revealed a significant difference among genotypes (F_2.28_ = 4.791, *p* = 0.016). Bonferroni post hoc analysis revealed a significant difference between RICH2^+/+^ and RICH2^−/−^ mice (*t* = 4.47, *p* = 0.043). **j** Significant genotype differences were found during rotarod test (two-way mixed ANOVA, genotype by trial interaction F_14.196_ = 2.202, *p* = 0.019; main effect of trial F_7.14_ = 16.845, *p* < 0.001, main effect of the genotype F_2.28_ = 3.025, *p* = 0.065). Post hoc analysis indicated a significant difference between RICH2^+/+^ and RICH2 ^−/−^ (*p* = 0.02) mice in the latency to fall of the rotarod. **e**–**j** Analysis and statistics were performed with *n* = 10 RICH2^+/+^, *n* = 12 RICH2^+/−^, *n* = 9 RICH2^−/−^ mice. Data are shown as mean, + SEM

Finally, we assessed the behavior of RICH2^−/−^ mice using several read-outs designed to evaluate behavior related to locomotion and activity (Additional file 3: Fig. S3a-e), anxiety and depression (Additional file 3: Fig. S3e-p), ASD-like behavior (Fig. 5i, Additional file 4: Fig. S4a-g), and learning and memory (Fig. 5a-d and i, Additional file 4: Fig. S4h-m). We did not detect differences in general health and neurological reflexes tested by a SHIRPA test (Additional file 5: Tab. S1). Since no significant difference in forepaw (chi-square: 1.216, df = 2, *p* = 0.526; Kruskal Wallis ANOVA) as well as forepaw and hindpaw grip strength (F_2.28_ = 1.216; *p* = 0.311; one way ANOVA) (Additional file 3: Fig. S3a and b) was detected, differences in setups requiring locomotion and activity are not expected to be caused by altered muscle strength. RICH2^−/−^ mice displayed normal general locomotor activity in the open field assay compared to RICH2^+/−^, and their wild type littermate controls, although RICH2^−/−^ mice tended to be less active (indicated by measures of distance traveled (F_2.28_ = 2.628, *p* = 0.096; one way ANOVA) and velocity (F_2.28_ = 2.555, *p* = 0.090; one way ANOVA)) (Additional file 3: Fig. S3c, d).

Differences in entries into and the time spent in the center zone vs. border zones might indicate anxiety related behavior in the open field test. However, no significant differences between genotypes in the number of ambulations (F_2.28_ = 0.507, *p* = 0.608), duration in boarder zone (F_2.28_ = 0.441 *p* = 0.648), duration in center zone (F_2.28_ = 0.025, *p* = 0.975), and entries into center zone (F_2.28_ = 1.696, *p* = 0.202) were observed (one way ANOVA) (Additional file 3: Fig. S3e-h). Similarly, we could not detect increased anxiety in an elevated plus maze (EPM) (Additional file 3: Fig. S3i-o). No significant difference in percent time spent in open arms (chi-square: 2.187, *df* = 2, *p* = 0.335; Kruskal-Wallis ANOVA), in the number of entries in open arms (F_2.28_ = 0.941, *p* = 0.402; one way ANOVA), number of entries into closed arms (F_2.28_ = 0.778, *p* = 0.469; one way ANOVA), and total number of entries (F_2.28_ = 0.929, *p* = 0.407; one way ANOVA) was detected (Additional file 3: Fig. S3i-l). Additionally, no significant difference (one way ANOVA) among genotypes was detected in activity related parameters such track length (F_2.28_ = 0.235, *p* = 0.792), velocity (F_2.28_ = 0.550, *p* = 0.583), and number of ambulations (F_2.28_ = 2.893, *p* = 0.073) (Additional file 3: Fig. S3m-o).

To investigate whether the trend observed towards hypo-locomotion in the open field and a decreased total number of ambulations in the EPM was based on a motivational problem, we performed a Porsolt Forced swim test (Additional file 3: Fig. S3p). However, we could not detect any significant difference among genotypes in the time spent immobile indicating no depression – like behavior (chi-square: 0.877, *df* = 2, *p* = 0.645; Kruskal-Wallis ANOVA).

Given that RICH2 is a direct interaction partner of SHANK3 in the PSD and that loss of SHANK3 has been linked to ASD in humans and animal models, we next evaluated RICH2^+/−^ and RICH2^−/−^ mice for ASD – like behavior (Fig.5i, Additional file 4: Fig. S4a-g). To that end, repetitive self-grooming was measured over a period of 10 min. RICH2^−/−^ mice display a significant increase in time spent self grooming in comparison to wild type mice (F_2.28_ = 4.791, *p* = 0.016; Bonferroni posttests analysis: *p* = 0.043) (Fig. 5i). Loss of fur due to excessive self-grooming was observed in some RICH2^−/−^ mice (Fig. 5i insert). Additionally, we assessed nesting behavior, but were unable to detect significant differences (Kruskal-Wallis analysis, chi-square: 1.133, *df* = 2, *p* = 0.567) (Additional file 4: Fig. S4a). Next, we performed an automated three chamber social approach task, to evaluate the animals for sociability and preference for social novelty (Additional file 4: Fig. S4b-g). RICH2^+/+^, RICH2^+/−^ and RICH2^−/−^ mice displayed normal sociability as each genotype preferred to spend significantly more time sniffing the wire cage containing a stranger mouse vs. the empty wire cage, with no effect of genotype (two-way mixed ANOVA, genotype by stimulus interaction F_2.28_ = 1.063, *p* = 0.359; stimulus main effect F_1.28_ = 130.248, *p* < 0.001; main effect of the genotype F_2.28_ = 1.151, *p* = 0.331; Post-tests of stimulus effect in all three genotypes: RICH2^+/+^*p* = 0.001; RICH2^+/−^*p* = 0.001; RICH2^−/−^*p* = 0.002) (Additional file 4: Fig. S4b). Further, all mice irrespective of genotype spend significant more time in the chamber containing a novel mouse than at the empty wire cage (two-way mixed ANOVA, genotype by stimulus interaction F_2.28_ = 0.349, *p* = 0.708; stimulus main effect F_1.28_ = 19.186, *p* < 0.001; main effect of the genotype F_2.28_ = 0.459, *p* = 0.636; Post-tests of stimulus effect in all three genotypes: RICH2^+/+^*p* = 0.05; RICH2^+/−^*p* = 0.027; RICH2^−/−^*p* = 0.016) (Additional file 4: Fig. S4c). Likewise, each genotype preferred sniffing the wire cage with the novel mouse (stranger 2) compared to the wire cage with the already known mouse (stranger 1) (two-way mixed ANOVA, genotype by stimulus interaction F_2.28_ = 0.557, *p* = 0.579; stimulus main effect F_1.28_ = 28.307, *p* < 0.001; main effect of the genotype F_2.28_ = 0.835, *p* = 0.444; Post-tests of stimulus effect in all three genotypes: RICH2^+/+^*p* = 0.029; RICH2^+/−^*p* = 0.01; RICH2^−/−^*p* = 0.018) (Additional file 4: Fig. S4d). In addition, there was a significant preference for the time spent in the chamber containing the stranger 2 in mice of all genotypes (two-way mixed ANOVA, genotype by stimulus interaction F_2.28_ = 0.163, *p* = 0.850; stimulus main effect F_1.28_ = 21.557, *p* < 0.001; main effect of the genotype F_2.28_ = 3.010, *p* = 0.065) (Additional file 4: Fig. S4e). No significant differences were detected in the number of transitions between genotypes during the sociability phase (two-way mixed ANOVA, genotype by stimulus interaction F_2.28_ = 1.715, *p* = 0.198; stimulus main effect F_1.28_ = 0.072, *p* = 0.790; main effect of the genotype F_2.28_ = 0.847, *p* = 0.439) and the social novelty task (two-way mixed ANOVA, genotype by stimulus interaction F_2.28_ = 1.185, *p* = 0.320; stimulus main effect F_1.28_ = 5.864, *p* = 0.022; main effect of the genotype F_2.28_ = 3.395, *p* = 0.048; Post-tests of stimulus effect in all three genotypes: RICH2^+/+^*p* = 0.470; RICH2^+/−^*p* = 0.432; RICH2^−/−^*p* = 0.081; main effect of genotype RICH2^+/−^ vs RICH2^−/−^*p* = 0.044) (Additional file 4: Fig. S4f and g).

To determine, whether knock-out of RICH2 impairs hippocampal dependent memory, we analyzed behavioral performance of RICH2^−/−^ and RICH2^+/−^ in various memory tests, including Y-maze, Morris Water Maze task, as well as novel object recognition and novel object location task.

In the Y-maze labyrinth, a hippocampus dependent task of spatial working memory, no significant difference between genotypes was detected in the percentage of alternation (Kruskal-Wallis ANOVA; chi-square: 2.717, *df* = 2, *p* = 0.257) and the number of entries (F_2.28_ = 2.053, *p* = 0.147; one way ANOVA) during the 5 min test session in the Y maze labyrinth (Additional file 4: Fig. S4h and i). Spatial memory was further tested using a Morris water maze (Additional file 4: Fig. S4j-m). During the acquisition training of the visible platform test, all three genotypes showed similar learning curves over 3 days, which indicates that RICH2^+/−^ as well as RICH2^−/−^ have no gross abnormalities in vision, swim ability and motivation to escape the water (two-way mixed ANOVA, genotype by trial interaction F_4.56_ = 1.946, *p* = 0.147; main effect of the trial F_2.56_ = 111.035, *p* < 0.001; main effect of the genotype F_2.28_ = 0.591, *p* = 0.560; Post-tests of the trial effect: RICH2^+/+^*p* < 0.001; RICH2^+/−^*p* < 0.001; RICH2^−/−^*p* < 0.001) (Additional file 4: Fig. S4j). In the invisible platform test, mice must learn the spatial relationships between objects in the room and the position of the platform to escape the water. During the acquisition training of the invisible platform test, no differences among genotypes were detected over 6 days (two-way mixed ANOVA, genotype by trial interaction F_10.140_ = 0.437, *p* = 0.901; main effect of the trial F_5.140_ = 11.357, *p* < 0.001; main effect of the genotype F_2.28_ = 0.919, *p* = 0.411; Post-tests of the trial effect: RICH2^+/+^ p < 0.001; RICH2^+/−^*p* = 0.002; RICH2^−/−^*p* = 0.005) (Additional file 4: Fig. S4k). Given that the mean track length was not altered amog genotypes (two-way mixed ANOVA, genotype by trial interaction F_10.140_ = 0.292, *p* = 0.982; main effect of the trial F_5.140_ = 9.179, *p* < 0.001; main effect of the genotype F_2.28_ = 0.648, *p* = 0.531; Post-tests of the trial effect: RICH2^+/+^*p* = 0.03; RICH2^+/−^*p* = 0.009; RICH2^−/−^*p* = 0.007) (Additional file 4: Fig. S4l), a possible memory deficit was not compensated by increased swimming activity. In the probe trial, all genotypes spend more time in the training quadrant, however not significant (RICH2^+/+^: (F_3.60_ = 0.391, *p* = 0.76); RICH2^+/−^: (F_3.30_ = 0.808, *p* = 0.500); RICH2^−/−^: (F_3.57_ = 0.513, p = 0.675)) (Additional file 4: Fig. S4m). Taken together, RICH2^−/−^ mice show normal spatial memory.

In contrast, RICH2^−/−^ mice show exaggerated responses to novel objects, but normal object recognition and location short-term memory (Fig. 5a-g). No innate preference for object positions was detected among all three genotypes (two-way mixed ANOVA, genotype by target interaction F_2.21_ = 0.569, *p* = 0.575; target main effect F_1.21_ = 2.792, *p* = 0.110; main effect of the genotype F_2.21_ = 1.319, *p* = 0.289). No significant differences were found between genotypes in the time the mice spent exploring the objects during different stages of the task for short-term memory. All genotypes showed a significant preference for the novel object (two-way mixed ANOVA, genotype by target interaction F_2.21_ = 1.252, *p* = 0.306; target main effect F_1.21_ = 37.531, *p* < 0.001; Post-tests of target effect in all three genotypes: RICH2^+/+^*p* = 0.006; RICH2^+/−^*p* = 0.007; RICH2^−/−^*p* = 0.034) during the test phase of the novel object recognition test for short term memory, indicating no impairment of short term novel object recognition memory (Fig. 5a).

Similarly, in the test for long-term recognition memory no innate preference for object position was detected (two-way mixed ANOVA, genotype by target interaction F_1.15_ = 0.009, *p* = 0.926; target main effect F_1.15_ = 0.098, *p* = 0.758). Wild type animals as well as RICH2^+/−^ showed a significant preference for the novel object in the test for long-term memory (two-way mixed ANOVA, genotype by target interaction F_1.15_ = 0.412, *p* = 0.531; target main effect F_1.15_ = 28.305, *p* < 0.001; Post-tests of target effect in genotypes: RICH2^+/+^*p* = 0.016; RICH2^+/−^*p* = 0.003). No conclusion can be drawn on RICH2^−/−^ since mice which explored objects less than 10 s were excluded from analysis (Fig. 5b) and only two RICH2^−/−^ met the criteria for inclusion in the analysis. This behavior, however, reveals a specific phenotype or RICH2^−/−^ animals (see below). In the test for spatial working memory, again, all tested genotypes showed a significant preference for the “moved” object during the test session (two-way mixed ANOVA, genotype by target interaction F_2.17_ = 0.575, *p* = 0.236; target main effect F_1.17_ = 90.745, *p* < 0.001; Post-tests of target effect in genotypes: RICH2^+/+^*p* = 0.001; RICH2^+/−^*p* = 0.001; RICH2^−/−^*p* = 0.028). No initial side preference was detected during the training session (two-way mixed ANOVA, genotype by target interaction F_2.17_ = 5.662, *p* = 0.013; target main effect F_1.17_ = 0.035, *p* = 0.986; Post-tests of genotype x target interaction: RICH2^+/+^*p* = 0.529; RICH2^+/−^*p* = 0.087; RICH2^−/−^*p* = 0.078) (Fig. 5c).

Tracking the path of mice before and after the presentation of novel objects during the novel object recognition task as well as novel location task (Fig. 5d) revealed marked differences in the response of RICH2^−/−^ to the presentation of a novel object during novel object - as well as novel location memory tests. When a novel object was placed in the open field arena during the training and test session of the test, a significant difference among genotypes was detected (two-way mixed ANOVA, genotype and test session interaction F_4.56_ = 2.557, *p* = 0.049; main effect of the genotype F_2.28_ = 10.613, *p* < 0.001; main effect of the test session F_2.56_ = 13.607, *p* = 0.001). Analyses reveal that RICH2^−/−^ mice displayed a significant preference for the non-object zone during the identical object presentation (two-way mixed ANOVA, genotype and zone interaction F_2.28_ = 8.608, *p* = 0.001; main effect of the zone F_1.28_ = 16.567, *p* = 0.372: main effect of the genotype F_2.28_ = 0.016, *p* = 0.984) (RICH2^−/−^*p* = 0.01) and the novel object presentation (two-way mixed ANOVA, genotype and zone interaction F_2.28_ = 8.744, *p* = 0.001; main effect of the zone F_1.28_ = 10.795, *p* = 0.003: main effect of the genotype F_2.28_ = 0.131, *p* = 0.878) (RICH2^−/−^*p* = 0.011), which was not present during the habituation phase, where no objects were present in the arena (two-way mixed ANOVA, genotype and zone interaction F_2.28_ = 2.544, *p* = 0.097; main effect of the zone F_1.28_ = 1.044, *p* = 0.316: main effect of the genotype F_2.28_ = 0.718, *p* = 0.497) (RICH2^−/−^*p* = 0.492). This exaggerated response to novel objects is also demonstrated comparing freezing behavior as well as locomotor activity before and after object - presentation (Fig. 5f and g). A significant difference in freezing response was detected between genotypes (two-way mixed ANOVA, genotype by test session interaction F_4.56_ = 3.280, *p* = 0.04; main effect of test session F_2.56_ = 0.478, *p* = 0.623, main effect of the genotype F_2.28_ = 5.452, *p* = 0.01). Post-tests analysis revealed a significantly increased time spent freezing of RICH2^−/−^ mice during the identical object presentation (RICH2^+/+^ vs RICH2^−/−^*p* = 0.019; RICH2^+/−^ vs RICH2^−/−^*p* = 0.011) and novel object presentation (RICH2^+/+^ vs RICH2^−/−^*p* = 0.012; RICH2^+/−^ vs RICH2^−/−^*p* = 0.017), which was not present during the habituation phase (Fig. 5f). Further, a significant genotype difference was found measuring track length comparing different phases of the test (two-way mixed ANOVA, genotype by test session interaction F_4.56_ = 1.739, *p* = 0.194; main effect of test session F_2.56_ = 8.612, *p* = 0.003, main effect of the genotype F_2.28_ = 7.399, *p* = 0.003) Post-test revealed that RICH2^−/−^ displayed a reduction in track length during identical object presentation (RICH2^+/+^ vs RICH2^−/−^*p* = 0.002, RICH2^+/−^ vs RICH2^−/−^*p* = 0.023) as well as novel object presentation (RICH2^+/+^ vs RICH2^−/−^*p* = 0.002, RICH2^+/−^ vs RICH2^−/−^*p* = 0.01) (Fig. 5g).

Thus, RICH2^−/−^ mice display active avoidance or anxiety towards novel objects. To determine whether the avoidance behavior towards novel objects is a general phenotype of reduced exploratory behavior, the amount of rearings was determined in a novel environment (Fig. 5h). However, no significant difference was found in the number of rearings in RICH2^−/−^ mice compared to wild type (F_2.26_ = 0.083, *p* = 0.920).

Additionally, RICH2 knock-out leads to impairments in motor learning (two-way mixed ANOVA, genotype by trial interaction F_14.196_ = 2.202, *p* = 0.019; main effect of trial F_7.14_ = 16.845, *p* < 0.001, main effect of the genotype F_2.28_ = 3.025, *p* = 0.065) Post-test analysis reveals a significant difference between RICH2^+/+^ and RICH2^−/−^ mice (*p* = 0.02) (Fig. 5j).

## Discussion

Here, we generated a RICH2^−/−^ mouse model to investigate the role of RICH2 at synapses *in vivo* and to evaluate the disruption of a suggested SHANK3/RICH2-complex at glutamatergic synapses. Three splice variants of RICH2 of 2000 bp, 2237 bp, and 2462 bp in size were found on the transcript level. On protein level, two putative RICH2 molecules appeared at 110 kDa and at 120 kDa in wild type mice. Our data shows a specific RICH2 knock-out of all isoforms in the brain of RICH2 mice by several experimental approaches.

The Rho GTPases are a large sub group of small GTP binding proteins among which RhoA, RAC1 and CDC42 are the most extensively studied members. The activity of these Rho GTPases is tightly regulated via the actions of several GAP, GEF and GDI proteins at each check point. In an *in vitro* study, it was shown that RICH2 is able to promote GTP hydrolysis and thus is able to theoretically inactivate RhoA, RAC1 and CDC42 [24]. However, among these three molecules, RAC1 forms a complex with RICH2 in mouse brain and thereby knock-out of RICH2 might exert its effect via the disinhibition of RAC1. Although the specificity of RhoGAP proteins in vivo may differ from the in vitro situation, given that many GAP proteins were reported to possess altered specificity towards GTPases in vivo and in vitro [11], we could confirm increased activation of CDC42 and in particular RAC1 in RICH2^−/−^ mice.

Generation and maturation of synapses requires the formation of dendritic spines, a process which is based upon actin rearrangements. Most of the studies analyzing the role of Rho GTPases on dendritic spine alteration have been done in vitro. It has been found that RhoA, RAC1 and CDC42 are able to regulate dendritic spine formation and morphological plasticity in specific ways. RhoA inhibits dendritic arbor growth, spine formation and maintenance whereas RAC1 and CDC42 promote such activity [4].

Since Rho GTPases are known to be modulators of actin dynamics within dendritic spines, we investigated whether RICH2 can be placed in a known small GTPase signaling pathway. RAC1 activity is essential for activity-dependent spine enlargement [26, 27], which is realized by modulation of actin polymerization [28]. In line with this, the over-expression of RICH2 and RAC1 have the opposite effect on dendritic spine size, where RICH2 overexpression decreased the spine size and RAC1 over-expression lead to an increase. Increased activation of RAC1 by knock-down of RICH2 has also been reported in Caco-2 epithelial cells [29].

Here, we could show that RICH2 knock-out results in an increase in actin levels in hippocampal S2 lysates and synapses of cultured neurons from RICH2^−/−^. Furthermore, we could show increased synaptic actin polymerization in RICH2^−/−^ mice.

RAC1 can affect actin dynamics via several pathways like activation or phosphorylation of PAK1 and LIMK, which in turn can inactivate the actin depolymerizing molecule coffilin [30]. Alternatively, RAC1 acts in a complex with ESP8 and IRSp53 [31]. RAC1 activation controls and also is controlled by EPS8/IRSp53 [31]. The absence of RICH2 leading to the over-activation of RAC1 results in altered Eps8 levels in RICH2^−/−^ mice. However, phosphorylation levels of PAK1 and LIMK were unchanged. A possible explanation is that the effect of RICH2 deletion alters actin dynamics not via LIMK1, but by increasing the amount of EPS8 promoting the formation of dendritic spines [25]. It has been shown that EPS8 can directly bind to F-actin or interact with E3b1 and Sos-1, regulating the morphology of excitatory synapses regarding spine size and dendritic shaft length, without altering the total number of spines [25], similarly to the phenotype observed in RICH2^−/−^ mice.

Intriguingly, it was shown that knock-down of SHANK3 in cell culture also leads to modifications of actin-dynamics in dendritic spines [32]. SHANK proteins in mature neurons compete with EPS8 to block actin bundling [33], antagonizing the actin bundling activity of IRSp53, which can be induced by EPS8 [33]. Thus, both the decrease in SHANK3 and disinhibition of RAC1 seen in RICH2^−/−^ mice might be facilitators of actin polymerization and may contribute to altered spine morphology.

Indeed, we found that RICH2 knock-out is accompanied by morphological abnormalities of spines. We have observed an enlargement of spines, without altering the number of spines in hippocampus and cerebellum. Along with these morphological changes detected by analysis of Golgi stained neurons, we found the number of spines with multiple PSDs increased using electron microscopy. The formation of multiple PSDs per spine may explain the increase in PSDs seen without an increase in spine numbers. However, given that previous studies indicated that disruption of the RICH2/SHANK3 complex inhibits dendritic spine enlargement [12] in vitro, further investigations on the complex dynamics of post-synaptic actin regulation in vivo might be needed to fully elucidate the role of Shank3 in this process, which might be isoform -, brain region -, and activity - dependent.

In addition, RICH2 knock-out alters the composition of synapses in the hippocampus and less so in the cerebellum. In particular RICH2^−/−^ mice show an increased expression of GluA4, GluN1, and GluN2A with a trend towards a reduction of GluN2B receptors. This increase was not based on higher gene transcription but might reflect a redistribution of soluble protein towards the PSD or a higher number of PSDs according to the quantification of mRNA.

Dendritic spine number and morphological abnormalities have been associated with neurological and psychiatric disorders like fragile X mental retardation syndrome [34]. Several protein regulating RAC1 such as TIAM1 (T-lymphoma invasion and metastasis-inducing protein 1) and DISC1 (Disrupted in schizophrenia 1) have been associated with psychiatric disorders in genome-wide association studies and the Neurexin-Neuroliginsynaptic complex that is highly linked to SHANK3 [35, 36] found to regulate the schizophrenia-related DISC1/KAL-7/RAC1 “Signalosome” [37].

In line with this, on behavioral level, the deletion of RICH2 resulted in specific abnormalities. RICH2^−/−^ mice showed impaired motor learning, which was not based on a general muscle weakness or motivational problem, an increase in stereotypic behavior such as self - grooming, and reduced exploration. The deficit in exploratory behavior was based on a specific fear of novel objects. Neither was exploratory behavior in general decreased nor anxiety in general increased in RICH2^−/−^ animals. A specific fear of novel objects has been rarely reported before in mouse animal models. Transgenic mice showing changes in neuroserpin levels, for example, were reported to show object neophobia but also increased anxiety [38]. Given that RICH2^−/−^ mice also show an increase in stereotypic behavior and that specific phobias such as irrational fear of objects and abnormal motor behavior have been reported in ASD patients, the behavior of RICH2 mice indeed shows some traits of ASD like behavior. Object neophobia might also reflect a stress response due to an increased need for sameness. However, while the behavioral deficits and the molecular interaction with SHANK3, a known autism associated gene, provide a link to ASD, we did not find alterations in other behavioral tests indicative for ASD. Thus, RICH2^−/−^ mice display a very specific subset of behavioral alterations visible in ASD mouse models, which might be caused by the differences in expression levels of SHANK3/RICH2 or RAC1 between brain regions and cell types [39].

The specific behavioral alterations observed in RICH2^−/−^ mice indicate the dysfunction of particular brain regions involved in the behavioral tasks. Intact motor learning, for example, is highly dependent on a functional cerebellum [40], while the fear of novel objects was indicated to be associated with hippocampal defects [41], but also occurs in animal models with cerebellar defects [42]. Furthermore, altered self-grooming can be, among other brain regions, associated with hippocampal dysfunction [43]. Indeed, cerebellum and hippocampus were the brain regions showing highest RICH2 expression.

## Conclusions

Taken together, we have seen that deletion of RAC1 GAP protein RICH2 leads to increases in spine volume and the number of spines with multiple head. Thus, our studies are in line with a deactivating function of RICH2 on RAC1 knowing the fact that conditional knock-down of RAC1 reduces spine density [34]. From our qRT PCR, WB and GTPase assay we have seen that the expression level of RAC1in RICH2^−/−^ mice is neither increased on transcriptional nor translational level, but the percentage of active GTP bound RAC1was increased. This activation could well be the underlying cause of the alteration of dendritic spine morphology, glutamatergic receptor localization at synaptic sites and actin polymerization. Thus, this study places RICH2 in the RAC1 signaling pathway at dendritic spines and provides a link between RAC1 signaling and the major organizers of the PSD, PSD-95 and SHANK3 proteins. In this complex RICH2 might be one of the critical control proteins regulating spine morphogenesis.

As several behavioral and functional abnormalities have been associated with mutations in Rho GTPases and their controlling molecules, further studies should focus on the SHANK3-RICH2-RAC1 complex as a putative target for therapeutic intervention.

## Methods

### Chemicals and reagents

Primary antibodies were purchased from Abcam (RICH2, PSD-95, CamKIIα/β, pCamKIIα/β (specific for the ~50 kDa αCamKII subunit and the ~60 kDa βCamKII subunit phosphorylated at Thr286), F-Actin), Abgent (RICH2), Cytoskeleton (RhoA, RAC1, CDC42); Sigma (GluN1, SHANK1, β-Actin), Alomone (GluA1, GluN2A); Millipore (GluN1, GluN2B, mGluR5, Cortactin); Synaptic Systems (GluA2, GluA3, GluA4, Calbindin), Novus Biologicals (GAPDH), Cell Signalling (PAK1, pPAK1, LIMK1, pLIMK1), Chemicon (WAVE1), and Santa Cruz (EPS8). SHANK3 antibodies have been described previously [44]. Secondary Alexa-coupled antibodies were purchased from Invitrogen. Unless otherwise indicated, all other chemicals were obtained from Sigma-Aldrich.

### Western blot-analysis

Western blot experiments were performed using PSD-enriched P2-fractions and cytosolic S2-fractions of distinct brain regions. Brain regions (hippocampus and cerebellum) of 3 wild type, 3 heterozygous and 3 knock-out mice (P70-P80) were thawed and 10 ml HEPES buffer per gram tissue was added (10 mM HEPES; 0.32 M Sucrose, pH 7.42; Protease Inhibitor Cocktail tablet and PhosSTOP phosphatase inhibitor (Roche, Germany)). Tissues were homogenized using sonication to obtain the CCH-fraction (crude cellular homogenate). To dissociate the nuclear fraction (P1), the CCH-lysate was centrifuged at 3200 rpm for 15 min at 4 °C. The resulting supernatant (S1) was centrifuged at 11400 rpm for 20 min at 4 °C. The pellet (P2; synaptosomal membrane fraction) was re-suspended in ice - cold HEPES buffer. Bradford-analysis was performed to measure protein concentrations. 10 μg of total protein was loaded onto PAGE in 4x SDS-sample buffer.

### Quantitative Real-time PCR

Isolation of total RNA from 3 mice per group was performed using the RNeasy kit (Qiagen) as described by the manufacturer. Isolated RNA was stored in RNase-free water at −80 °C. Thermal cycling and fluorescent detection were performed using the Rotor-Gene-Q real-time PCR machine (model 2-Plex HRM) (Qiagen) and QuantiFast SYBR Green RT-PCR kit. The qRT-PCR was assayed in 0.1 ml strip tubes in a total volume of 20 μl reaction mixture. The SYBR Green I reporter dye signal was measured against the internal passive reference dye (ROX) to normalize non-PCR-related fluctuations in. Data were analyzed using the hydroxymethylbilane synthase (HMBS) gene to normalize transcript levels. Cycle threshold (ct) values were calculated by the Rotor- Gene-Q Software (version 2.0.2). All reactions were run in technical triplicates and mean ct values for each reaction were taken for data analysis.

### Cloning

For the myc-RICH2: *mouse ARHGAP44 transcript variant 2/*pCMV myc (C2) plasmid, forward and reverse primers were flanked by HindIII (*fwd*) and EcoRI (*rev*) restriction sites, the insert amplified using a PCR-mix (Promega GoTaq Green), and cloned into a pCMV MYC (Clontech) vector.

### Hippocampal cultures from rat or mouse brain

The preparation of hippocampal cultures was performed essentially as described previously [45]. After preparation, hippocampal neurons (embryonic day 18; E18) were seeded on poly-L-lysine (0.1 mg/ml; Sigma-Aldrich) coated glass coverslips. Cells were grown in Neurobasal medium (Invitrogen), complemented with B27 supplement (Invitrogen), 0.5 mM L-Glutamine (Invitrogen) and 100 U/ml penicillin/streptomycin (Invitrogen) and maintained at 37 °C in 5 % CO_2_.

### Immunocytochemistry

Cultured cells were fixed with 4 % paraformaldehyde (PFA) and 4 % sucrose in phosphate-buffered saline (PBS) at RT for 15 min. Cells were permeabilized with 0.2 % Triton X-100 in PBS for 10 min. Then, blocking was performed using 10 % FCS in PBS for 2 h followed by incubation with the primary antibody in PBS at 4 °C overnight. 3× 5 min washing with PBS was followed by incubation with the secondary antibody (dilution 1:500) for 1 h at RT. Cell nuclei were stained with DAPI (AppliChem, Darmstadt) followed by washing with water. Cover slips were mounted using VectaMount AQ (Vector Labs).

### Histology

Immunohistochemistry - Cryosections were thawed for 20 min and fixed in paraformaldehyde (PFA) for 20 min in a hydrated box. After fixation, sections were washed 3× 5 min with PBS followed by permeabilization with 0.2 % Triton in PBS for 1 h. Later the slices were washed again 3× 10 min with PBS containing 0.05 % Triton and blocked with 10 % FCS in PBS for 2 h. After blocking, sections were incubated with the primary antibody overnight at 4 °C followed by 10 min washing with PBS containing 0.05 % Triton. Then the sections were incubated with the secondary antibody at 37 °C for 2 h. After washing 3× 15 min with PBS containing 0.05 % Triton, sections were washed 5 min with PBS containing DAPI and after a final washing step in ddH_2_O, slices were mounted with VectaMount (Vector Laboratories).

Nissl staining - Brains were removed from animals and snap frozen and kept at −80 °C until further use. Brain tissue was sectioned with 16 μm thickness using a Cryotome and embedded in OCT (Sakura Finetek, USA). For Nissl staining, slices were hydrated in 1 % w/v Cresyl Violet (Merck Millipore) for 5 min. The slices were subjected to dehydration using gradient ethanol and finally cleaned with xylene.

Golgi staining - Golgi staining was performed on brain cryosections from WT and KO animals using the FD Rapid GolgiStain Kit (FD NeuroTechnologies) according to the manufacturer's instructions.

For Nissl and Golgi staining, images were taken with a Mirax Scanner (Carl Zeiss, Germany) and analyzed using Pannoramic Viewer and ImageJ. In each set of KO and WT animals, brain slices from similar plane and region were stained and analyzed.

### Electron Microscopy (EM)

The animals were perfused with 0.9 % NaCl with 0.5 ml Heparin/100 ml solution and fixed with 2 % PFA, 2.5 % Glutaraldehyde, 1 % Sucrose, 0.1 M Cacodylate buffer solution. The brains were removed and kept in 2.5 % Glutaraldehyde, 1 % Sucrose, 0.1 M Cacodylate buffer solution overnight. Next day, the brains were washed with 0.1 M Cacodylate buffer with 1 % Sucrose for 1 h. Brains were sliced with 200 μM thickness and the desired region (i.e. cerebellum) was cut from the whole brain. The samples were further cut and processed in the Facility for Electron Microscopy, University of Ulm and analyzed by transmission electron microscope EM 10 (Zeiss) at 80 kV and ImageJ software.

### GTPase Assay

GTPase assays for RhoA, RAC1 and CDC42 were performed on P2 lysates of brain tissues from WT and KO animals using the RHOA / RAC1 / CDC42 G-LISA Activation Assay Bundle 3 Kits (BK 135, Cytoskeleton) according to the manufacturer's instructions and protocol. The absorbance was measured using a Tecan Infinite® 200 PRO microplate reader.

For detecting RAC1-GTP level in cultured hippocampal neuron, RAC1 G-LISA Activation Assay (Luminescence format, BK 126, Cytoskeleton) was used according to the manufacturer's instructions and protocol.

### Actin polymerization assay

Actin polymerization assays were performed with the P2 lysates of 3 wt and 3 KO animals using the Actin Polymerization Biochem kit from Cytoskeleton (Cat. BK 003) according to the manufacturer's instructions and protocol. The fluorescence was measured using a Fluoroskan Ascent® FL microplate reader.

### Electrophysiology

Electrophysiological experiments were performed with embryonic hippocampal neurons cultivated for 14 days. Membrane currents were recorded in the whole-cell recording mode using an EPC-9 amplifier and Patchmaster software (HEKA, Lambrecht, Germany) [46]. Before recording, cells were extensively washed in extracellular standard solution composed of (in mM): 145 NaCl, 5 KCl, 2 CaCl_2_, 25 glucose and 12 HEPES; pH 7.4, supplemented with strychnine (1 μM), bicuculline (10 μM) and tetrodotoxin (TTX; 50 nM). Patch pipettes were drawn from borosilicate glass with tip resistances between 2 and 3 MOhm when filled with (in mM) 140 CsCl_2_, 2 MgCl_2_, 2 ATPx2Na, 10 HEPES; pH 7.3. To improve sealing, tips were briefly dipped into 2 % dimethylsilane dissolved in dichlormethane. Unless otherwise stated all experiments were performed at room temperature and the membrane potential clamped to −80 mV.

### Animals and housing conditions

RICH2^−/−^ mice were generated by the integration of a gene-trap vector. RICH2^−/−^ ES cell lines were obtained from BayGenomics. All animal experiments were performed in compliance with the guidelines for the welfare of experimental animals issued by the Federal Government of Germany and approved by the local ethics committee at Ulm University (ID Number: 0.103 and 1146). For the generation of animals, heterozygotes were used for breedings. Mutants and controls were littermates.

Six week-old mice (RICH2^+/+^, RICH2^+/−^, RICH2^−/−^, backcrossed for more than 10 generations on C57BL/6 J background) were transferred from the animal facility and habituated for 10 days. All animals were housed individually upon arrival in plastic cages under standard laboratory conditions (maintained at 22 °C, with lights automatically turned on/off in a 12 h rhythm (lights on at 7 am)) and provided with food and water available *ad libitum*. Experiments were preformed between 9 am and 6 pm. Prior to the behavioral experiments, mice were habituated for 1 h to the test room. Only male mice were used for behavioral testing.

### Behavioral phenotyping

Behavioral tests were conducted in the following order (1) General health and neurological reflexes, (2) Grip Strength Test, (3) Nest pattern in the home cage, (4) Self grooming, (5) Elevated Plus Maze, (6) Open Field, (7) Accelerated Rotarod, (8) Y Maze, (9) Sociability and Social Novelty, (10) Novel Object Recognition for Short and Long Term Memory, (11) Novel Object Location, (12) Morris Water Maze Acquisition, and (13) Porsolt Forced Swim Test.

#### General health and neurological reflexes

The mice were evaluated for general health and neurological reflexes using modified SHIRPA Test protocol [47], with exception of the startle response. Additionally, mice were observed for the visual placing reflex and for the ability to grasp a metal grip with forepaws and hindpaws. Muscle grip strength was measured using a Bioseb Grip Meter (Bioseb, France) on forelimbs and all limbs. The test was repeated three consecutive times for each mouse.

Self grooming - Mice were scored for spontaneous grooming behaviors. Observations and recordings were made in a soundproof room under dim red light (8 lux), mounted 100 cm above the cage. Each mouse was placed into a standard mouse cage (26.5 cm × 20 cm × 14 cm), filled with a thin layer of 1 cm of fresh bedding in order to prevent digging. A video camera (Conrad CCD camera S/W) was mounted approximately 15 cm in front of the test cage. An inclined mirror was positioned behind the test cage to include the mice facing away from the camera. After habituation for 15 min, each mouse was scored with a stopwatch for accumulative time grooming each body region for a period of 10 min.

#### Accelerated rotarod performance

Mice were placed on a rotarod apparatus (TSE Systems, Bad Homburg, Germany) with 4 rpm for 30 s for habituation. Subsequently, the rotational speed was increased from 4 to 40 rpm within 5 min. Mice were given 4 trials with 45 min break between each trial per day. Mice were tested on 2 consecutive days for a total amount of 6 trials. The latency to fall off the rod was measured. Mice that fall of the rod in less than 10 s were given a second trial.

#### Novel object recognition

The test was conducted in the open field arena (50 × 50 cm) and consisted of three phases: habituation, acquisition and retention. Mice were first habituated to the open field arena (without any object inside) for 30 min and placed back into the home-cage for approximately 1–2 min. In the meantime two identical copies of the same objects were placed in the two corners of the open field arena, approximately 4 cm from the sidewall. The mouse was placed back into the same arena facing the opposite side of the objects and allowed to freely explore the setting for 10 min. After this acquisition period, the mouse was placed back into the home cage. Following various retention intervals (10 min for short term memory, 24 h for long term memory) the subject mouse was transferred back to the arena where one of the identical objects was replaced by a novel object. The mouse was allowed to explore the setting for 10 min. Recorded videos were scored manually with a stopwatch. Object exploration was defined as a clear nose contact with the object. In order to measure recognition memory, a preference index for the novel object was calculated as the ratio of the time spent exploring the novel object over the total time spent exploring both objects. (Discrimination index, DI = Novel object exploration x 100/(Novel object exploration + old object exploration). Activity parameters (distance moved, and time spent in the object zone vs time spent in the non object zone) and the time spent freezing during all oft the three different test sessions (habituation, training and test phase) was scored using the video tracking software EthoVision XT (Noldus, Wageningen, Netherlands). Freezing behavior was determined with the mobility parameter of EthoVision XT (immobility threshold: 1 %). Mice which displayed object exploration of less than 10 s were excluded from data analysis.

#### Novel object location

After a 30 min habituation phase in the open field arena, mice were exposed to two identical objects, which were placed in the left and right boarder zone of the open field arena (approximately 4 cm). The mice were allowed to freely explore the objects during a sample phase of 10 min. After this acquisition period, mice were placed back in the home cage. Following a delay of 10 min, the position of one object was changed to the opposite corner of the open field arena. Time spent exploring the object that has changed position was compared with time spent exploring the object in the old location. Analysis and recording was preformed as described above. The location of the moved object was counter-balanced between mice.

Nest pattern in the home cage - One week before the test, a nestlet (5 × 5 cm, mean weight 2.6 g) was introduced in the home cage of mice in order to prevent neophobia and facilitate habituation to the nestlet. The nesting material remained undisturbed in the home cage until the test day. At the test day, 2 h before the dark phase, the old nest material was removed and a new nestlet was placed in the home cage of the mouse. Nest building ability was assessed the following morning, according to a 5 point rating scale [48].

#### Locomotion

Exploratory activity and locomotion in a novel environment was assessed by a 30 min test session in an open field arena. The arena was evenly illuminated by overhead white lighting (100 lux) and constructed of white Plexiglas. The tested mouse was placed in the center of a 50 × 50 cm open field arena (with 20 × 20 cm center zone) and allowed to freely explore for 30 min period. Average velocity, total distance traveled, and the time spent in the center zone vs. time spent at the border zone, number of entries into center zone and boarder zone, as well as ambulations was measured using Viewer 2 software (Bioserve GmbH, Bonn, Germany).

#### Elevated Plus Maze for anxiety-like behavior

Anxiety-related behaviors were assessed using the elevated-plus maze paradigm, that is based on the natural aversion of mice to avoid height and open spaces. The apparatus consists of two open and two enclosed (with 16 cm high walls) horizontal perpendicular arms (30 × 5 cm) positioned at 60 cm above the floor. The junction of the four arms creates a central square platform (5 × 5 cm). Every mouse was placed in the central platform, facing one of the closed arms and allowed to explore the setting for 10 min. The behavior of every mouse was analyzed using Viewer 2 software (Bioserve GmbH, Bonn, Germany) in terms of time spent and entries in open arms and closed arms, average velocity and ambulations.

#### Sociability and social novelty

Social approach and preference for social novelty was tested in an apparatus that consisted of a rectangular three-chambered box and retractable doorways within the two dividing walls as previously described by Yang et al*.* [49]. A video camera was mounted (Conrad CCD camera S/W) over rectangular chamber to allow recording of the sessions. Videos were digitized by Pinnacle Studio 500-PCI, version 10. Between each subject the chamber was cleaned with 70 % ethanol and wiped with dry paper and left 5 min in order to allow ethanol evaporation. Observations and recordings were made in a soundproof room. The test consisted of three different sessions: habituation, sociability and social novelty. Habituation: In order to facilitate acclimation before the sociability session, the subject mouse was first placed in the middle chamber (doors open) and allowed to freely explore the whole setting, in which both side compartments contained an empty wire cup for a period of 10 min (session 1). Sociability: After this habituation period, the subject mouse was briefly confined in the central compartment, while an unfamiliar C57BL/6 mouse of the same sex (male) and age (stranger 1) was placed under one of the cups. Between each trial the location of the stranger 1 (left vs. right side) was systematically alternated. After the placement of stranger 1, the doors were simultaneously re-opened and the tested mouse was then allowed to explore the whole apparatus for 10 min (session 2). Preference for social novelty: At the end of the 10 min sociability test, the test mouse was again restricted to the central compartment while another unfamiliar C57BL/6 mouse of the same sex (male) (stranger 2) was placed under the other wire cage. The tested mouse could then again freely explore the whole apparatus for 10 min (session 3). In all three phases, measures were taken of the amount of time spent in each chamber, number of entries into each chamber by using the program EthoVision XT (Noldus, Wageningen, Netherlands). An entry was defined as the center point of the mouse was in one of the 3 chambers. For the time spent sniffing, each wire cage was manually scored by a human observer with a stopwatch. Sniffing was defined as a clear nose contact with the wire cage. The animals serving as stranger mice, were adult male C57BL/6 J aged matched and obtained from Janvier Labs. Stranger mice had no previous physical contact with the test mouse and were kept in a separate location from the subject mice.

#### Y-maze

Continuous, spontaneous alternation behavior (SAB), was assessed in a symmetrical Y Maze (3 arms, 40 x 9 cm with 16 cm high walls), a hippocampal dependent task of spatial working memory. Arms choices (all four paws entering one arm) were recorded, while mice were allowed to freely explore the Y-shaped labyrinth for a period of 5 min. Alternation was determined by recording the order of the visited arms (A, B or C). Overlapping triplets of 3 arm visits was counted as one complete spontaneous alternation. The SAB score was calculated after following formula: (number of spontaneous alternation)/(total number of arm visits −2). In order to prevent odor traces between animals, the walls and bottom of the Y Maze were carefully cleaned with 70 % ethanol and wiped out with clean with paper towels. Videos were recorded by and analyzed using Viewer 2 software (Bioserve GmbH, Bonn, Germany).

#### Morris water maze

The apparatus consisted of a circular pool (120 cm diameter, 45 cm deep), partially filled with milky water (22-24 °C, 45 cm deep). The maze was located in a room containing several visual cues, evenly illuminated by overhead white lighting (110 lux). Mice were trained for their ability to find an escape platform (diameter: 12 cm) on five different components (visible platform acquisition, hidden platform acquisition, probe trial, hidden platform for reversal learning, probe trial for reversal learning). Visible platform (non spatial learning): The visible platform test was conducted on 2 consecutive days, with four trials per day. Mice were trained to swim to an escape platform that was marked by a 15 cm patterned cylinder extending above the water surface. The landmark cues were hidden during these trials. For each trial the mouse was placed on one of the four possible randomly assigned start location. If a mouse found the platform within 60 s the trial ended and the animal was allowed to remain on the platform for further 20 s. After 20 s the next trial was started.

Hidden platform test (spatial learning): After the visible platform test was preformed, mice were trained to learn the location of a submerged platform by external landmarks that were placed at the walls of the testing room. The training was conducted for 8 consecutive days (4 trials per day, inter-trial interval 5 min). Any mouse that failed to navigate to the platform within 90 s was manually guided to the platform. The animal remained 20 s on the platform before being removed from the pool.

Probe trial: After 8 consecutive days of training, a probe trial was preformed in order to determine if mice used spatial navigation to find the platform. In this case, the platform was removed from the pool and the mouse was allowed to swim freely for 90 s in the pool. The percentage of time the mouse spent in each quadrant of the pool was calculated, as well as the number of times the mice crossed the former position of the hidden platform.

#### Forced swim test

The Porsolt Forced swim test was conducted in a transparent glass cylinder (diameter 25 cm) filled to a depth of 15 cm with water (24 - 26 °C) water. A video camera (Conrad CCD camera S/W) was mounted approximately 15 cm in front of the test cage to allow recordings from the lateral view. Mice were gently placed in the water for a period of 6 min. Immobility was defined as a complete lack of limb movement, with exception of movements necessary to keep the mouse afloat. The duration of immobility was scored by an experienced observer during the last 4 min of the 6 min test period using UleadVideoSoftware version 7.0 (accuracy 40 ms).

### Statistics

#### Signal intensities

Fluorescence images were obtained using an upright Axioscope microscope equipped with a Zeiss CCD camera (16 bits; 1280 × 1024 pixels per image) using the AxioVision software (Zeiss) with the same exposure time throughout the experiment and all of the conditions, and were analyzed using ImageJ 1.49i. Background fluorescence was determined and signals 10 % above background fluorescence measured. Synaptic fluorescence intensity for all immunoreactive puncta for a single neuron in a single image was measured excluding axo-somatic synapses. The mean and SEM were determined from these data points and from 10 cells per condition. Statistical analysis was performed using Microsoft Excel for Macintosh and tested for significance using unpaired *t*-tests. For the comparison for more than two groups, one way ANOVA was performed. All values were normally distributed.

#### Western blot quantification

Images of bands were taken using a MicroChemi 4.2 imaging device (Biostep) with GelCapture version 2.0 software. Western blot bands were quantified using ImageJ. All WB bands were normalized to GAPDH and the ratios averaged and tested for significance using unpaired *t*-tests.

#### Behavior

RICH2^+/+^, RICH2^+/−^ and RICH2^−/−^ littermate controls were compared for each behavioral task. First normal distribution was determined by Shapiro-Wilk-test. Genotype differences in general health and neurological reflexes, grip strength, open field, elevated plus-maze, forced swim test, number of rearings, self grooming, nest building and spontaneous alternation in the Y- Maze, were analyzed for parametric data using one-way Analysis of Variance (ANOVA). Significant ANOVA results were followed by Bonferroni post hoc test. Non parametric data were examined using Kruskal-Wallis ANOVA test. Significant Kruskal-Wallis ANOVA results were followed by individual post hoc group comparison using the Mann–Whitney U adjusted for multiple comparisons. For the automated three chamber social approach task, rotarod performance, freezing response, track length response toward objects, Morris water maze, and novel object recognition test and novel object location, data were analyzed for parametric data using two-way mixed ANOVA, followed by post-hoc tests. For the three chamber test, time spent in the center is depicted on the graphs for illustrative purpose only.

Statistical analysis was preformed with SPSS version 20. Statistical tests were two tailed with a significance level of α ≤ 0.05. Statistically significant differences are indicated in the Figures by * *p* ≤ 0.05, ** *p* ≤ 0.01 and *** *p* ≤ 0.001.

## Additional file

Additional file 1:
**Figure S1.** Characterization of RICH2 antibodies and mice. a) Western Blot analysis showing RICH2 immunoreactivity in purified subcellular fractions (CCH *crude cellular homogenate*, S1 *supernatant 1*, P1 *nuclear fraction*, S2 *cytosolic fraction*, *P2 synaptoneurosomes*) extracted from whole brains of wild type, heterozygous and knock-out mice (P70) to be ubiquitously present in analyzed fractions. RICH2 immunoreactive bands consistently disappeared in the knock-out mouse lysate using two different antibodies. b) Immunoblots using cortical P2-fractions to verify functionality of five different RICH2 antibodies (Abcam rb-αRICH2 (*ab93627*), ABGENT rb-αRICH2 (*AP10656b)*, rb-αRICH2 serum1, rb-αRICH2 serum2, gp-αRICH2 serum). All antibodies detected a band at 120 kDa that was present in the wild type lysate but could not be detected in the knock-out lysate. c) Genomic situation in wild type vs. knock-out mice. Recombination events during random gene-trap vector insertion generated a 46.7 kb chromosomal deletion 3’ of the gene trap vector from exon 2 up to and including exon 6 (genomic DNA (grey), deleted genomic sequence and gene-trap insertion (red)). RT-PCR approaches using specific sense primers for the gene-trap insertion paired with exon 7 specific antisense primers show the presence of the gene-trap insertion located just next to the 5’- end of exon 7. d) Offspring from breeding of heterozygous mice did not deviate from the expected Mendelian distribution (0.25:0.50:0.25). (TIF 1850 kb) Additional file 2:
**Figure S2.** Cellular characterization of RICH2^−/−^ mice. a) Electron microscopy of immunogold stained RICH2 showing the protein to be located at the PSD. b) Pulldown experiment from RICH2/SHANK3 co-transfected cells using αRICH2 serum antibody showed a clear interaction between RICH2 and SHANK3 as known from previous studies. c) RICH2 immunostaining of primary hippocampal neurons (DIV14) cultured from wild type (+/+) and RICH2 knock-out (−/−) E18 embryos. No RICH2 immunoreactive signal can be seen in RICH2^−/−^ cultures. d) Quantification of GTP-bound RAC1 in RICH2^−/−^ normalized against wild type hippocampal neurons (DIV14) confirms the finding of increased RAC1 activity in hippocampal brain tissue. A significant increase in RAC1-GTP was found (unpaired *t*-test, df = 4, *p* = 0.0001, *t* = 16.6757). e) Quantification of synaptic signal intensities of Synaptophysin1 and RICH2 in primary hippocampal neurons (DIV14). While no change in Synaptophysin1 levels occurs (unpaired *t*-test, df = 8, *p* = 0.3094, *t* = 1.0853), RICH2 signals are significantly decreased (absent) from dendrites and synaptic sites in knock-out animals (df = 8, *p* = 0.0001, *t* = 14.2878). The average signal intensity of RICH2 (co-localizing with Synaptophysin1) signals from 5 cells is shown. f) MAP2 immunocytochemical staining of DIV14 hippocampal neurons from wild type and knock-out E18 mouse embryos. Analysis of dendritic branching by counting primary, secondary, tertiary and quaternary dendrites reveals no significant differences between wild type and knock-out cells (unpaired *t*-test, primary: df = 4, *p* = 0.0824, *t* = 2.3062; secondary: df = 4, *p* = 0.8492, *t* = 0.2027; tertiary: df = 4, *p* = 0.1475, *t* = 1.7925; quaternary: df = 4, *p* = 0.96, t = 0.0534; total: df = 4, *p* = 0.3333, *t* = 1.0995). g) Spontaneous miniature excitatory currents were recorded from wild type (+/+) (*n* = 20) and RICH2 knock-out (−/−) (*n* = 12) hippocampal neurons at DIV14. A trend towards an increased mEPSC amplitude was found in RICH2^−/−^ cells (unpaired *t*-test, df = 30, *p* = 0.1733, *t* = 1.3949). The mean signal area is significantly increased in RICH2^−/−^ cells (df = 30, *p* = 0.0492, *t* = 2.05). No significant alterations between RICH2^+/+^ and RICH2^−/−^ cells could be found regarding mEPSC frequencies, and no difference was detected in rise and decay times (unpaired *t*-test, frequency: df = 30, *p* = 0.4494, *t* = 0.7665; rise time: df = 30, *p* = 0.5133, *t* = 0.6615; decay time: df = 30, *p* = 0.2637, *t* = 1.1391). h) The mean fluorescence intensity and signal area (i) of actin and Homer1b/c co-localizing immunoreactive puncta was measured from 10 hippocampal neurons from wild type (+/+) and RICH2^−/−^ mice at DIV14. Actin and Homer1b/c signal intensities are increased in RICH2^−/−^ mice (unpaired *t*-test, Actin: df = 18, *p* = 0.0005, *t* = 4.1906; Homer1: df = 18, *p* = 0.0103, *t* = 2.8653) and a trend towards an increase of synaptic actin signal area compared to wild type mice can be seen (unpaired *t*-test, df = 18, *p* = 0.0993, *t* = 1.738). Exemplary images (right panel) show additional DAPI stained nuclei (blue) in merged images. (TIF 2986 kb) Additional file 3:
**Figure S3.** Behavioral analysis of RICH2^−/−^ mice I. a, b) Muscle strength was measured on forelimbs and all limbs. No significant differences was detected in forepaw (a) (chi-square: 1.216, df = 2, *p* = 0.526; Kruskal-Wallis ANOVA) as well as forepaw and hindpaw grip strength (b) (F_2.29_ = 1.216, *p* = 0.311; one way ANOVA). c-h) Open field test: General locomotor activity in the open field assay across a 30 min test session in male RICH2^−/−^, RICH2^+/−^, and their wild type littermate controls. c) Distance travelled, d) velocity, e) number of ambulations, f) duration in boarder zone, g) duration in center zone, h) entries into center zone. Although RICH2^−/−^ mice tended to be less active, no significant differences between genotypes were found in the secondary parameters of the open field test. One way ANOVA analysis revealed a trend toward hypo-locomotion as indicated by distance traveled (c) (F_2.28_ = 2.628, *p* = 0.096), the primary dependent variable in the open field test, and velocity (d) (F_2.28_ = 2.555, *p* = 0.090). Direct comparison of mutant and wild type revealed no significant difference. Similarly, one way ANOVA revealed no significant difference between genotypes in the number of ambulations (e) (F_2.28_ = 0.507, *p* = 0.608), duration in boarder zone (f) (F_2.28_ = 0.441 *p* = 0.648), duration in center zone (g) (F_2.28_ = 0.025, *p* = 0.975), and entries into center zone (h) (F_2.28_ = 1.696, *p* = 0.202). i-o) Elevated plus maze performance of male RICH2^−/−^, RICH2^+/−^ and RICH2^+/+^ mice during a 10 min test session. i) Percent time spent in open arms, j) number of entries into open arms, k) number of entries into closed arms, l) total number of entries, m) track length, n) velocity, and o) number of ambulations. Overall RICH2^−/−^ mice show no anxiety related behavior in the paradigm of the elevated plus maze. The Kruskal-Wallis ANOVA analysis revealed no significant difference in percent time spent in open arms among genotypes (i) (chi-square: 2.187, df = 2, *p* = 0.335). Additionally, no significant difference was detected (one way ANOVA) in the number of entries in open arms (j) (F_2.28_ = 0.941, *p* = 0.402), number of entries into closed arms (k) (F_2.28_ = 0.778, *p* = 0.469), and total number of entries (l) (F_2.28_ = 0.929, *p* = 0.407). Likewise, no significant difference (one way ANOVA) among genotype was detected in activity parameters, track length (m) (F_2.28_ = 0.235, *p* = 0.792), velocity (n) (F_2.28_ = 0.550, *p* = 0.583), and number of ambulations (o) (F_2.27_ = 2.893, *p* = 0.073). p) Depression - related behavior in the Porsolt Forced swim test. Kruskal-Wallis ANOVA Analysis revealed no significant difference among genotypes in the time spent immobile (chi-square: 0.877, df = 2, *p* = 0.645). a-p) All analyses and statistics were performed with *n* = 10 RICH2^+/+^, *n* = 12 RICH2^+/−^, *n* = 9 RICH2^−/−^ mice. Data are shown as mean, ± SEM. (TIF 1001 kb) Additional file 4:
**Figure S4.** Behavioral analysis of RICH2^−/−^ mice II. RICH2^+/−^ and RICH2^−/−^ mice were analyzed and compared to wild type littermates. Using several paradigms, ASD-like behavior (a-g), and learning and memory (h-m) were assessed. a) Nesting behavior: Histogram of average nest score taken 24 h after a nestlet was introduced in the home cage. No significant difference among RICH2^+/+^, RICH2^+/−^ and RICH2^−/−^ mice was present in nest quality scores (Kruskal-Wallis analysis, chi-square: 1.133, df = 2, *p* = 0.567). b-g) Sociability and preference for social novelty scores in the automated three chambered social approach task. b) Normal sociability was found in all genotypes (RICH2^+/+^, RICH2^+/−^ and RICH2^−/−^). All genotypes spend significantly more time sniffing the wire cage containing a stranger mouse vs. the empty wire cage (two-way mixed ANOVA, genotype by stimulus interaction F_2.28_ = 1.063, *p* = 0.359; stimulus main effect F_1.28_ = 130.248, *p* < 0.001; main effect of the genotype F_2.28_ = 1.151, *p* = 0.331; Post-tests of stimulus effect in all three genotypes: RICH2^+/+^ *p* = 0.001; RICH2^+/−^ *p* = 0.001; RICH2^−/−^ *p* = 0.002). c) Similarly, all genotypes demonstrated a significant preference for spending time in the side containing the stranger mouse, versus time in the chamber with the empty wire cage (two-way mixed ANOVA, genotype by stimulus interaction F_2.28_ = 0.349, *p* = 0.708; stimulus main effect F_1.28_ = 19.186, *p* < 0.001; main effect of the genotype F_2.28_ = 0.459, *p* = 0.636; Post-tests of stimulus effect in all three genotypes: RICH2^+/+^ *p* = 0.05; RICH2^+/−^ *p* = 0.027; RICH2^−/−^ *p* = 0.016). d) In the second part of the test however, while in the social novelty task, all genotypes still show a significant preference for stranger 2, indicated as time spent sniffing the wire cage (two-way mixed ANOVA, genotype by stimulus interaction F_2.28_ = 0.557, *p* = 0.579; stimulus main effect F_1.28_ = 28.307, *p* < 0.001; main effect of the genotype F_2.28_ = 0.835, *p* = 0.444; Post-tests of stimulus effect in all three genotypes: RICH2^+/+^ *p* = 0.029; RICH2^+/−^ *p* = 0.01; RICH2^−/−^ *p* = 0.018). e) There was no significant preference for the time spent in the chamber containing the stranger 2 in RICH2^−/−^ mice (two-way mixed ANOVA, genotype by stimulus interaction F_2.28_ = 0.163, *p* = 0.850; stimulus main effect F_1.28_ = 21.557, *p* < 0.001; main effect of the genotype F_2.28_ = 3.010, *p* = 0.065). f, g) No significant differences were detected in the number of transitions between genotypes during the sociability (f) sociability (two-way mixed ANOVA, genotype by stimulus interaction F_2.28_ = 1.715, *p* = 0.198; stimulus main effect F_1.28_ = 0.072, *p* = 0.790; main effect of the genotype F_2.28_ = 0.847, *p* = 0.439) and social novelty task (g) (two-way mixed ANOVA, genotype by stimulus interaction F_2.28_ = 1.185, *p* = 0.320; stimulus main effect F_1.28_ = 5.864, *p* = 0.022; main effect of the genotype F_2.28_ = 3.395, *p* = 0.048; Post-tests of stimulus effect in all three genotypes: RICH2^+/+^ *p* = 0.470; RICH2^+/−^ *p* = 0.432; RICH2^−/−^ *p* = 0.081; main effect of genotype RICH2^+/−^ vs RICH2^−/−^ *p* = 0.044). h, i) Spontaneous alternation behavior in the Y-maze labyrinth, a hippocampus dependent task of spatial working memory. h) No significant difference between genotypes in the percentage of alternation during the 5 min test session in the Y maze labyrinth (Kruskal-Wallis ANOVA; chi-square: 2.717, df = 2, *p* = 0.257). i) Additionally, no significant difference in the number of entries between genotype (F_2.28_ = 2.053, *p* = 0.147; one way ANOVA) was detected. j-m) Learning and memory testing using the Morris Water Maze. j, k) Training trials for the visible as well as invisible platform. For each day of training, data were averaged across the four daily trials and in all two - way mixed ANOVAs, “day” was treated as the repeated measurement. j) During the acquisition training of the visible platform test, all three genotypes showed similar learning curves over 3 days (two-way mixed ANOVA, genotype by trial interaction F_4.56_ = 1.946, *p* = 0.147; main effect of the trial F_2.56_ = 111.035, *p* < 0.001; main effect of the genotype F_2.28_ = 0.591, *p* = 0.560; Post-tests of the trial effect: RICH2^+/+^ *p* < 0.001; RICH2^+/−^ *p* < 0.001; RICH2^−/−^ *p* < 0.001). k) During the acquisition training of the invisible platform test, all three genotypes showed similar learning curves over 6 days (two-way mixed ANOVA, genotype by trial interaction F_10.140_ = 0.437, *p* = 0.901; main effect of the trial F_5.140_ = 11.357, *p* < 0.001; main effect of the genotype F_2.28_ = 0.919, *p* = 0.411; Post-tests of the trial effect: RICH2^+/+^ *p* < 0.001; RICH2^+/−^ *p* = 0.002; RICH2^−/−^ *p* = 0.005). l) Track length was similar across genotypes (two-way mixed ANOVA, genotype by trial interaction F_10.140_ = 0.292, *p* = 0.982; main effect of the trial F_5.140_ = 9.179, *p* < 0.001; main effect of the genotype F_2.28_ = 0.648, *p* = 0.531; Post-tests of the trial effect: RICH2^+/+^ *p* = 0.03; RICH2^+/−^ *p* = 0.009; RICH2^−/−^ *p* = 0.007). m) In the probe trial, all genotypes spent more time in the training quadrant (T: Target, AL: Adjacent left, O: Opposite), however not significant (RICH2^+/+^: (F_3.60_ = 0.391, *p* = 0.76); RICH2^+/−^: (F_3.30_ = 0.808, *p* = 0.500); RICH2^−/−^: (F_3.57_ = 0.513, *p* = 0.675), within group repeated measures ANOVA). a-m) All analyses and statistics were performed with *n* = 10 RICH2^+/+^, *n* = 12 RICH2^+/−^, *n* = 9 RICH2^−/−^ mice. Data are shown as mean, ± SEM. (TIF 1485 kb) Additional file 5:
**Table 1.** Behavioral analysis of RICH2^−/−^ mice. RICH2^+/−^ and RICH2^−/−^ mice were analyzed and compared to wild type littermates. General health (SHIRPA test): Control, RICH2^+/−^ and RICH2^−/−^ mice do not show significant differences in general health, motor coordination, locomotor activity or reflexes. (PDF 59 kb)

## Abbreviations

ASD: Autism spectrum disorder
CNS: Central nervous system
EPM: Elevated plus maze
IHC: Immunohistochemistry
PSD: Postsynaptic density
RhoSAP: RhoGAP synapse associated protein
SHANK3: SH3 and multiple ankyrin repeat domains 3

## References

[1] Boeckers TM (2006). The postsynaptic density. Cell Tissue Res.

[2] Verpelli C, Schmeisser MJ, Sala C, Boeckers TM (2012). Scaffold proteins at the postsynaptic density. Adv Exp Med Biol.

[3] Tada T, Sheng M (2006). Molecular mechanisms of dendritic spine morphogenesis. Curr Opin Neurobiol.

[4] Newey SE, Velamoor V, Govek EE, Van Aelst L (2005). Rho GTPases, dendritic structure, and mental retardation. J Neurobiol.

[5] Koh CG (2005). Rho GTPases and their regulators in neuronal functions and development. Neurosignals.

[6] Tashiro A, Yuste R (2008). Role of Rho GTPases in the morphogenesis and motility of dendritic spines. Methods Enzymol.

[7] Haditsch U, Leone DP, Farinelli M, Chrostek-Grashoff A, Brakebusch C, Mansuy IM (2009). A central role for the small GTPase Rac1 in hippocampal plasticity and spatial learning and memory. Mol Cell Neurosci.

[8] Fiorentini C, Falzano L, Travaglione S, Fabbri A (2003). Hijacking Rho GTPases by protein toxins and apoptosis: molecular strategies of pathogenic bacteria. Cell Death Differ.

[9] Tcherkezian J, Lamarche-Vane N (2007). Current knowledge of the large RhoGAP family of proteins. Biol Cell.

[10] Van Aelst L, D'Souza-Schorey C (1997). Rho GTPases and signaling networks. Genes Dev.

[11] Richnau N, Aspenström P (2001). Rich, a rho GTPase-activating protein domain-containing protein involved in signaling by Cdc42 and Rac1. J Biol Chem.

[12] Raynaud F, Janossy A, Dahl J, Bertaso F, Perroy J, Varrault A (2013). Shank3-Rich2 interaction regulates AMPA receptor recycling and synaptic long-term potentiation. J Neurosci.

[13] Roussignol G, Ango F, Romorini S, Tu JC, Sala C, Worley PF (2005). Shank expression is sufficient to induce functional dendritic spine synapses in aspiny neurons. J Neurosci.

[14] Sala C, Piëch V, Wilson NR, Passafaro M, Liu G, Sheng M (2001). Regulation of dendritic spine morphology and synaptic function by Shank and Homer. Neuron.

[15] Kreienkamp HJ (2002). Organisation of G-protein-coupled receptor signalling complexes by scaffolding proteins. Curr Opin Pharmacol.

[16] Kreienkamp HJ (2008). Scaffolding proteins at the postsynaptic density: shank as the architectural framework. Handb Exp Pharmacol.

[17] Boeckers TM, Bockmann J, Kreutz MR, Gundelfinger ED (2002). ProSAP/Shank proteins - a family of higher order organizing molecules of the postsynaptic density with an emerging role in human neurological disease. J Neurochem.

[18] Boeckers TM, Winter C, Smalla KH, Kreutz MR, Bockmann J, Seidenbecher C (1999). Proline-rich synapse-associated proteins ProSAP1 and ProSAP2 interact with synaptic proteins of the SAPAP/GKAP family. Biochem Biophys Res Commun.

[19] Haeckel A, Ahuja R, Gundelfinger ED, Qualmann B, Kessels MM (2008). The actin-binding protein Abp1 controls dendritic spine morphology and is important for spine head and synapse formation. J Neurosci.

[20] Kuriu T, Inoue A, Bito H, Sobue K, Okabe S (2006). Differential control of postsynaptic density scaffolds via actin-dependent and -independent mechanisms. J Neurosci.

[21] Durand CM, Betancur C, Boeckers TM, Bockmann J, Chaste P, Fauchereau F (2007). Mutations of the synaptic scaffolding protein SHANK3 are associated with autism spectrum disorders. Nat Genet.

[22] Grabrucker AM, Schmeisser MJ, Schoen M, Boeckers TM (2011). Postsynaptic ProSAP/Shank scaffolds in the cross-hair of synaptopathies. Trends Cell Biol.

[23] Leblond CS, Nava C, Polge A, Gauthier J, Huguet G, Lumbroso S (2014). The Meta-analysis of SHANK Mutations in Autism Spectrum Disorders: A Gradient of Severity in Cognitive Impairments. Plos Genet.

[24] Raynaud F, Moutin E, Schmidt S, Dahl J, Bertaso F, Boeckers TM (2014). Rho-GTPase-activating protein interacting with Cdc-42-interacting protein 4 homolog 2 (Rich2): a new Ras-related C3 botulinum toxin substrate 1 (Rac1) GTPase-activating protein that controls dendritic spine morphogenesis. J Biol Chem.

[25] Stamatakou E, Marzo A, Gibb A, Salinas PC (2013). Activity-dependent spine morphogenesis: a role for the actin-capping protein Eps8. J Neurosci.

[26] Nakayama AY, Harms MB, Luo L (2000). Small GTPases Rac and Rho in the maintenance of dendritic spines and branches in hippocampal pyramidal neurons. J Neurosci.

[27] Tashiro A, Yuste R (2004). Regulation of dendritic spine motility and stability by Rac1 and Rho kinase: evidence for two forms of spine motility. Mol Cell Neurosci.

[28] Croisé P, Estay-Ahumada C, Gasman S, Ory S (2014). Rho GTPases, phosphoinositides, and actin: a tripartite framework for efficient vesicular trafficking. Small GTPases.

[29] Rollason R, Korolchuk V, Hamilton C, Jepson M, Banting G (2009). A CD317/tetherin-RICH2 complex plays a critical role in the organization of the subapical actin cytoskeleton in polarized epithelial cells. J Cell Biol.

[30] Yang N, Higuchi O, Ohashi K, Nagata K, Wada A, Kangawa K, Nishida E, Mizuno K (1998). Cofilin phosphorylation by LIM-kinase 1 and its role in Rac-mediated actin reorganization. Nature.

[31] Funato Y, Terabayashi T, Suenaga N, Seiki M, Takenawa T, Miki H (2004). IRSp53/Eps8 complex is important for positive regulation of Rac and cancer cell motility/ invasiveness. Cancer Res.

[32] Duffney LJ, Wei J, Cheng J, Liu W, Smith KR, Kittler JT (2013). Shank3 deficiency induces NMDA receptor hypofunction via an actin-dependent mechanism. J Neurosci.

[33] Sawallisch C, Berhörster K, Disanza A, Mantoani S, Kintscher M, Stoenica L (2009). The insulin receptor substrate of 53 kDa (IRSp53) limits hippocampal synaptic plasticity. J Biol Chem.

[34] Bongmba OY, Martinez LA, Elhardt ME, Butler K, Tejada-Simon MV (2011). Modulation of dendritic spines and synaptic function by Rac1: a possible link to Fragile X syndrome pathology. Brain Res.

[35] Bourgeron T (2009). A synaptic trek to autism. Curr Opin Neurobiol.

[36] Arons MH, Thynne CJ, Grabrucker AM, Li D, Schoen M, Cheyne JE (2012). Autism-associated mutations in ProSAP2/Shank3 impair synaptic transmission and neurexin-neuroligin-mediated transsynaptic signaling. J Neurosci.

[37] Owczarek S, Bang ML, Berezin V (2015). Neurexin-Neuroligin Synaptic Complex Regulates Schizophrenia-Related DISC1/Kal-7/Rac1 “Signalosome”. Neural Plast.

[38] Madani R, Kozlov S, Akhmedov A, Cinelli P, Kinter J, Lipp HP (2003). Impaired explorative behavior and neophobia in genetically modified mice lacking or overexpressing the extracellular serine protease inhibitor neuroserpin. Mol Cell Neurosci.

[39] Schmeisser MJ, Ey E, Wegener S, Kuebler A, Bockmann J, Shiban E (2012). Autistic-like behaviours and hyperactivity in mice lacking ProSAP1/Shank2. Nature.

[40] Shiotsuki H, Yoshimi K, Shimo Y, Funayama M, Takamatsu Y, Ikeda K (2010). A rotarod test for evaluation of motor skill learning. J Neurosci Methods.

[41] Bliss-Moreau E, Toscano JE, Bauman MD, Mason WA, Amaral DG (2010). Neonatal amygdala or hippocampus lesions influence responsiveness to objects. Dev Psychobiol.

[42] DeLorey TM, Sahbaie P, Hashemi E, Homanics GE, Clark JD (2008). Gabrb3 gene deficient mice exhibit impaired social and exploratory behaviors, deficits in non-selective attention and hypoplasia of cerebellar vermal lobules: a potential model of autism spectrum disorder. Behav Brain Res.

[43] Cannon RL, Paul DJ, Baisden RH, Woodruff ML (1992). Alterations in self-grooming sequences in the rat as a consequence of hippocampal damage. Psychobiology.

[44] Grabrucker S, Jannetti L, Eckert M, Gaub S, Chhabra R, Pfaender S (2014). Zinc deficiency dysregulates the synaptic ProSAP/Shank scaffold and might contribute to autism spectrum disorders. Brain.

[45] Grabrucker A, Vaida B, Bockmann J, Boeckers TM (2009). Synaptogenesis of hippocampal neurons in primary cell culture. Cell Tissue Res.

[46] Hamill OP, Marty A, Neher E, Sakmann B, Sigworth FJ (1981). Improved patch-clamp techniques for high-resolution current recording from cells and cell-free membrane patches. Pflugers Arch.

[47] Rogers DC, Fisher EM, Brown SD, Peters J, Hunter AJ, Martin JE (1997). Behavioral and functional analysis of mouse phenotype: SHIRPA, a proposed protocol for comprehensive phenotype assessment. Mamm Genome.

[48] Deacon RM (2006). Assessing nest building in mice. Nat Protocol.

[49] Yang M, Silverman JL, Crawley JN (2011). Automated three-chambered social approach task for mice. Curr Protoc Neurosci.

