# Supplementary figures and images for: Enlarged dendritic spines and pronounced neophobia in mice lacking the PSD protein RICH2
Source: Mol Brain. 2016 Mar 11;9:28. doi: 10.1186/s13041-016-0206-6 (PMC4788860; doi:10.1186/s13041-016-0206-6)

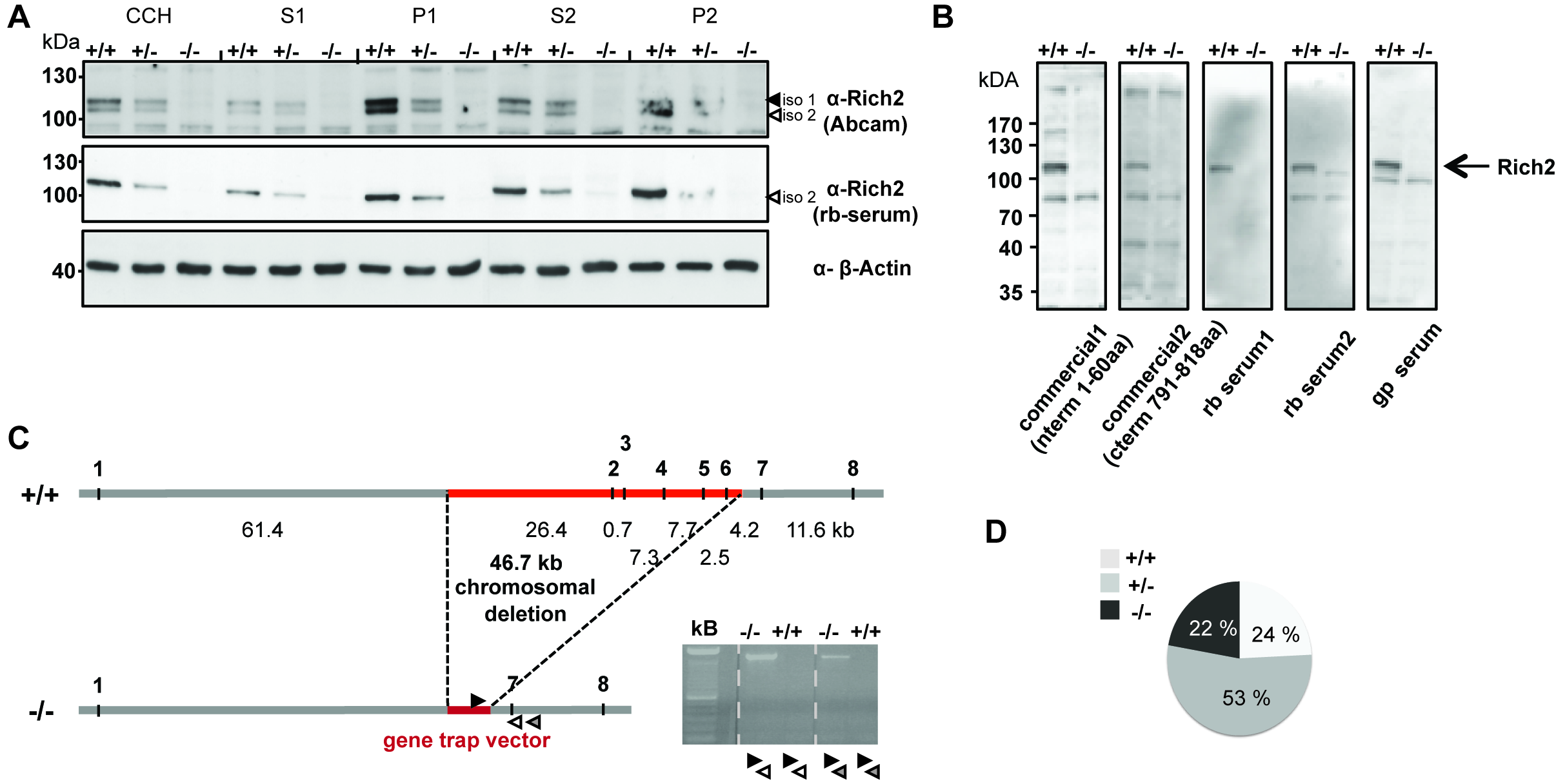

Supplement: Additional file 1: — Figure S1. Characterization of RICH2 antibodies and mice. a) Western Blot analysis showing RICH2 immunoreactivity in purified subcellular fractions (CCH crude cellular homogenate, S1 supernatant 1, P1 nuclear fraction, S2 cytosolic fraction, P2 synaptoneurosomes) extracted from whole brains of wild type, heterozygous and knock-out mice (P70) to be ubiquitously present in analyzed fractions. RICH2 immunoreactive bands consistently disappeared in the knock-out mouse lysate using two different antibodies. b) Immunoblots using cortical P2-fractions to verify functionality of five different RICH2 antibodies (Abcam rb-αRICH2 (ab93627), ABGENT rb-αRICH2 (AP10656b), rb-αRICH2 serum1, rb-αRICH2 serum2, gp-αRICH2 serum). All antibodies detected a band at 120 kDa that was present in the wild type lysate but could not be detected in the knock-out lysate. c) Genomic situation in wild type vs. knock-out mice. Recombination events during random gene-trap vector insertion generated a 46.7 kb chromosomal deletion 3’ of the gene trap vector from exon 2 up to and including exon 6 (genomic DNA (grey), deleted genomic sequence and gene-trap insertion (red)). RT-PCR approaches using specific sense primers for the gene-trap insertion paired with exon 7 specific antisense primers show the presence of the gene-trap insertion located just next to the 5’- end of exon 7. d) Offspring from breeding of heterozygous mice did not deviate from the expected Mendelian distribution (0.25:0.50:0.25). (TIF 1850 kb) [file 13041_2016_206_MOESM1_ESM.tif]

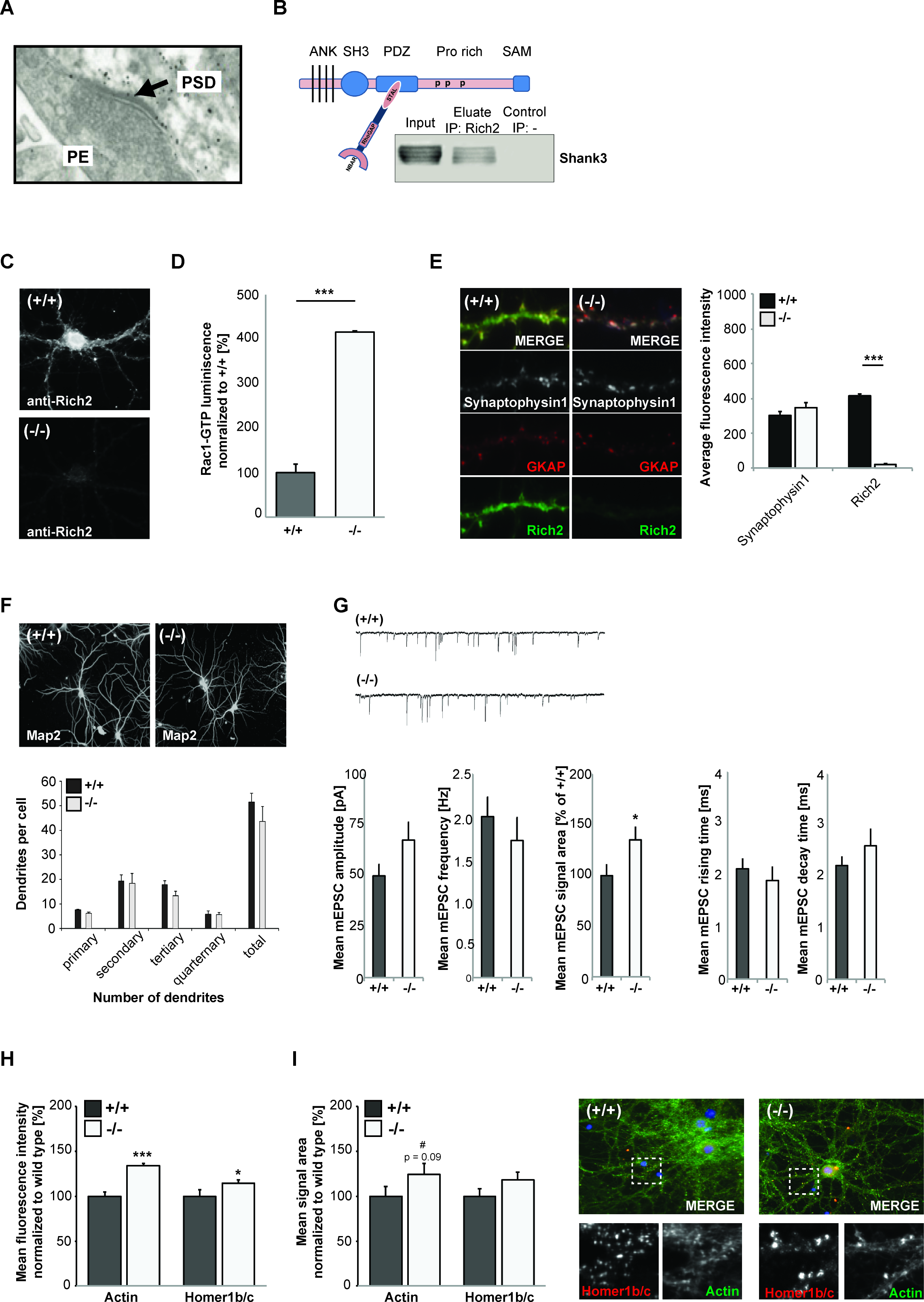

Supplement: Additional file 2: — Figure S2. Cellular characterization of RICH2−/− mice. a) Electron microscopy of immunogold stained RICH2 showing the protein to be located at the PSD. b) Pulldown experiment from RICH2/SHANK3 co-transfected cells using αRICH2 serum antibody showed a clear interaction between RICH2 and SHANK3 as known from previous studies. c) RICH2 immunostaining of primary hippocampal neurons (DIV14) cultured from wild type (+/+) and RICH2 knock-out (−/−) E18 embryos. No RICH2 immunoreactive signal can be seen in RICH2−/− cultures. d) Quantification of GTP-bound RAC1 in RICH2−/− normalized against wild type hippocampal neurons (DIV14) confirms the finding of increased RAC1 activity in hippocampal brain tissue. A significant increase in RAC1-GTP was found (unpaired t-test, df = 4, p = 0.0001, t = 16.6757). e) Quantification of synaptic signal intensities of Synaptophysin1 and RICH2 in primary hippocampal neurons (DIV14). While no change in Synaptophysin1 levels occurs (unpaired t-test, df = 8, p = 0.3094, t = 1.0853), RICH2 signals are significantly decreased (absent) from dendrites and synaptic sites in knock-out animals (df = 8, p = 0.0001, t = 14.2878). The average signal intensity of RICH2 (co-localizing with Synaptophysin1) signals from 5 cells is shown. f) MAP2 immunocytochemical staining of DIV14 hippocampal neurons from wild type and knock-out E18 mouse embryos. Analysis of dendritic branching by counting primary, secondary, tertiary and quaternary dendrites reveals no significant differences between wild type and knock-out cells (unpaired t-test, primary: df = 4, p = 0.0824, t = 2.3062; secondary: df = 4, p = 0.8492, t = 0.2027; tertiary: df = 4, p = 0.1475, t = 1.7925; quaternary: df = 4, p = 0.96, t = 0.0534; total: df = 4, p = 0.3333, t = 1.0995). g) Spontaneous miniature excitatory currents were recorded from wild type (+/+) (n = 20) and RICH2 knock-out (−/−) (n = 12) hippocampal neurons at DIV14. A trend towards an increased mEPSC amplitude was found in RICH2−/− cells [file 13041_2016_206_MOESM2_ESM.tif]

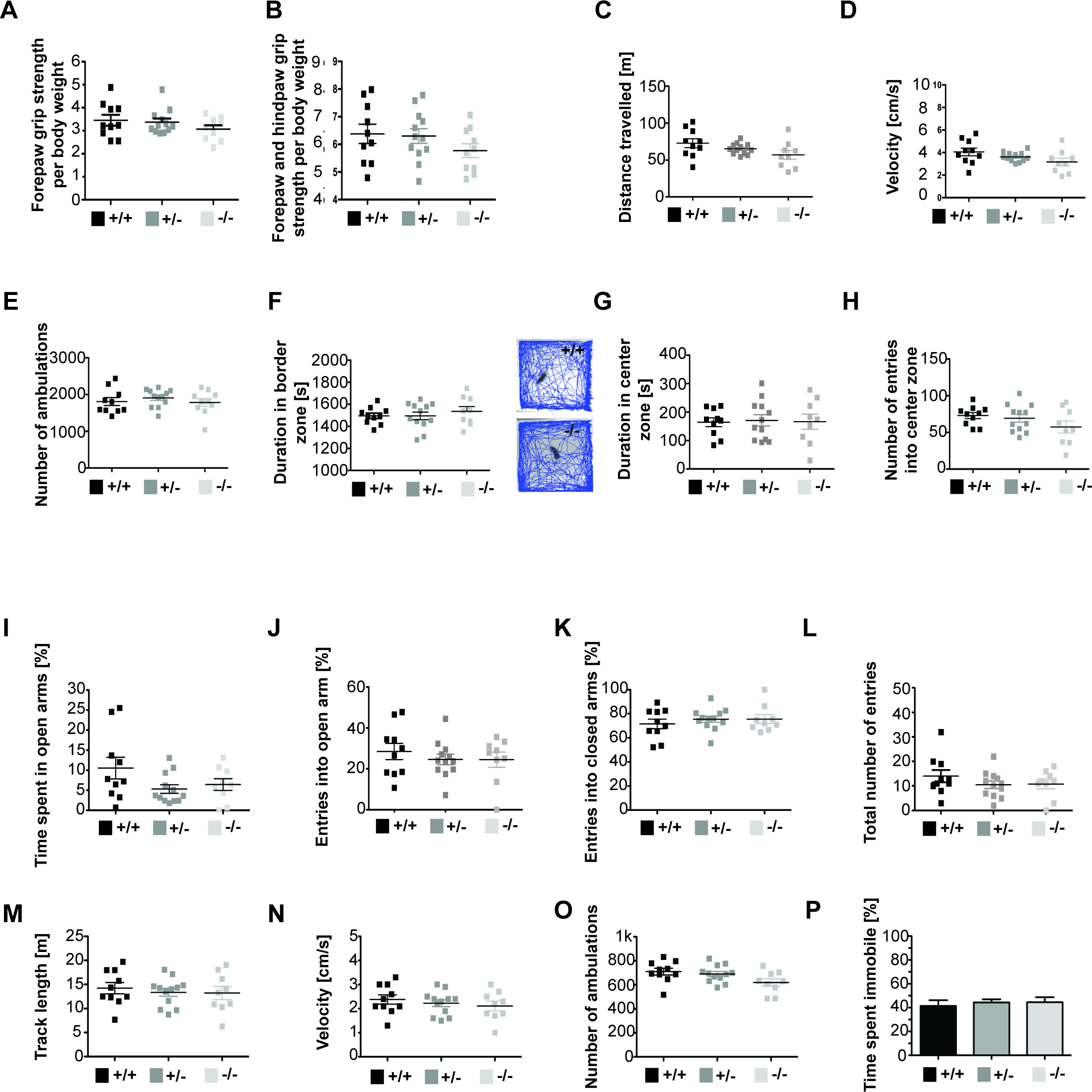

Supplement: Additional file 3: — Figure S3. Behavioral analysis of RICH2−/− mice I. a, b) Muscle strength was measured on forelimbs and all limbs. No significant differences was detected in forepaw (a) (chi-square: 1.216, df = 2, p = 0.526; Kruskal-Wallis ANOVA) as well as forepaw and hindpaw grip strength (b) (F2.29 = 1.216, p = 0.311; one way ANOVA). c-h) Open field test: General locomotor activity in the open field assay across a 30 min test session in male RICH2−/−, RICH2+/−, and their wild type littermate controls. c) Distance travelled, d) velocity, e) number of ambulations, f) duration in boarder zone, g) duration in center zone, h) entries into center zone. Although RICH2−/− mice tended to be less active, no significant differences between genotypes were found in the secondary parameters of the open field test. One way ANOVA analysis revealed a trend toward hypo-locomotion as indicated by distance traveled (c) (F2.28 = 2.628, p = 0.096), the primary dependent variable in the open field test, and velocity (d) (F2.28 = 2.555, p = 0.090). Direct comparison of mutant and wild type revealed no significant difference. Similarly, one way ANOVA revealed no significant difference between genotypes in the number of ambulations (e) (F2.28 = 0.507, p = 0.608), duration in boarder zone (f) (F2.28 = 0.441 p = 0.648), duration in center zone (g) (F2.28 = 0.025, p = 0.975), and entries into center zone (h) (F2.28 = 1.696, p = 0.202). i-o) Elevated plus maze performance of male RICH2−/−, RICH2+/− and RICH2+/+ mice during a 10 min test session. i) Percent time spent in open arms, j) number of entries into open arms, k) number of entries into closed arms, l) total number of entries, m) track length, n) velocity, and o) number of ambulations. Overall RICH2−/− mice show no anxiety related behavior in the paradigm of the elevated plus maze. The Kruskal-Wallis ANOVA analysis revealed no significant difference in percent time spent in open arms among genotypes (i) (chi-square: 2.187, df = 2, p = 0.335). Additional [file 13041_2016_206_MOESM3_ESM.tif]

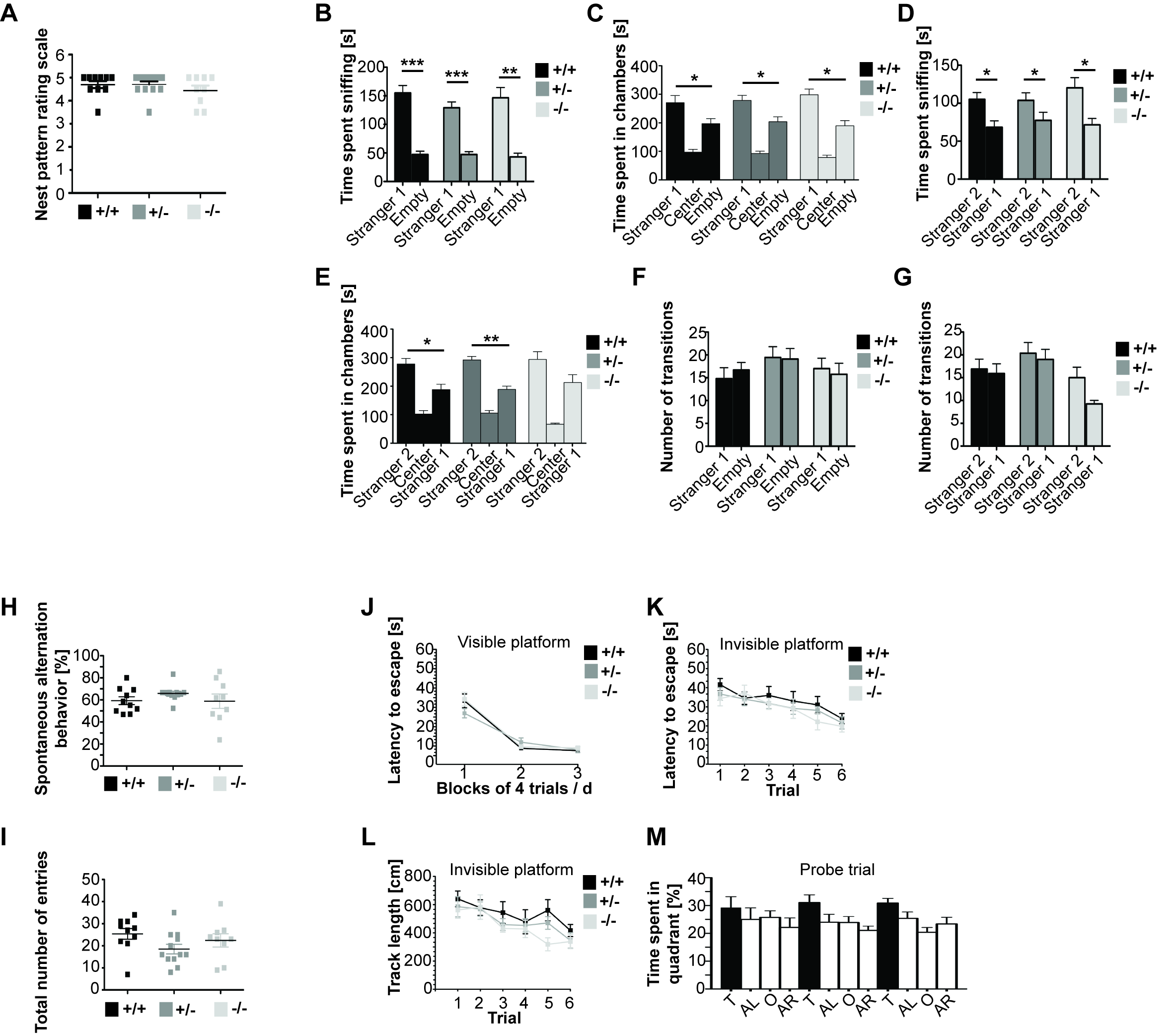

Supplement: Additional file 4: — Figure S4. Behavioral analysis of RICH2−/− mice II. RICH2+/− and RICH2−/− mice were analyzed and compared to wild type littermates. Using several paradigms, ASD-like behavior (a-g), and learning and memory (h-m) were assessed. a) Nesting behavior: Histogram of average nest score taken 24 h after a nestlet was introduced in the home cage. No significant difference among RICH2+/+, RICH2+/− and RICH2−/− mice was present in nest quality scores (Kruskal-Wallis analysis, chi-square: 1.133, df = 2, p = 0.567). b-g) Sociability and preference for social novelty scores in the automated three chambered social approach task. b) Normal sociability was found in all genotypes (RICH2+/+, RICH2+/− and RICH2−/−). All genotypes spend significantly more time sniffing the wire cage containing a stranger mouse vs. the empty wire cage (two-way mixed ANOVA, genotype by stimulus interaction F2.28 = 1.063, p = 0.359; stimulus main effect F1.28 = 130.248, p < 0.001; main effect of the genotype F2.28 = 1.151, p = 0.331; Post-tests of stimulus effect in all three genotypes: RICH2+/+ p = 0.001; RICH2+/− p = 0.001; RICH2−/− p = 0.002). c) Similarly, all genotypes demonstrated a significant preference for spending time in the side containing the stranger mouse, versus time in the chamber with the empty wire cage (two-way mixed ANOVA, genotype by stimulus interaction F2.28 = 0.349, p = 0.708; stimulus main effect F1.28 = 19.186, p < 0.001; main effect of the genotype F2.28 = 0.459, p = 0.636; Post-tests of stimulus effect in all three genotypes: RICH2+/+ p = 0.05; RICH2+/− p = 0.027; RICH2−/− p = 0.016). d) In the second part of the test however, while in the social novelty task, all genotypes still show a significant preference for stranger 2, indicated as time spent sniffing the wire cage (two-way mixed ANOVA, genotype by stimulus interaction F2.28 = 0.557, p = 0.579; stimulus main effect F1.28 = 28.307, p < 0.001; main effect of the genotype F2.28 = 0.835, p = 0.444; Post-tests of stimulus effe [file 13041_2016_206_MOESM4_ESM.tif]
