# Supplementary material for: Enlarged dendritic spines and pronounced neophobia in mice lacking the PSD protein RICH2
Source: Mol Brain. 2016 Mar 11;9:28. doi: 10.1186/s13041-016-0206-6 (PMC4788860; doi:10.1186/s13041-016-0206-6)
Supplement: Additional file 5: — Table 1. Behavioral analysis of RICH2−/− mice. RICH2+/− and RICH2−/− mice were analyzed and compared to wild type littermates. General health (SHIRPA test): Control, RICH2+/− and RICH2−/− mice do not show significant differences in general health, motor coordination, locomotor activity or reflexes. (PDF 59 kb) [file 13041_2016_206_MOESM5_ESM.pdf]

		Rich+/+	Rich+/-	Rich-/-	
number of animal (male mice)		(n=10)	(n=12)	(n=9)	**p-value**
weight [g]		26.27 (±0.74)	27.06 (±0.76)	27.41 (±0.8)	one way ANOVA ($F_{2.28}$ = 1.265, p = 0.298)
Body position	(0 =inactive, 1 = active, 2 = excessive active)	0.55 (±0.16)	0.45 (±0.16)	0.45 (±0.16)	one way ANOVA ($F_{2.28}$ = 0.082, p = 0.921)
Tremor	(0 = absent, 1 = present)	0	0	0	n.s.
Palpebral closure	(0 = eyes open, 1 = eyes closed)	0	0	0	n.s.
Coat appearance	(0 = tidy and well groomed coat, 1 = irregularities such as piloerection)	0	0	0	n.s.
Whiskers	(0 = present, 1 = absent)	0	0	0	n.s.
Defecation	(0 = present, 1 = absent)	0.1 (±0.12)	0.1 (±0.14)	0.27 (±0.13)	one way ANOVA ($F_{2.28}$ = 0.516, p = 0.602)
Transfer arousal	(0 = extended freeze (over 5 seconds), 1 = briefly freeze followed by movement, 2 = immediate movement)	0.89 (±0.26)	0.76 (±0.23)	0.81 (±0.26)	one way ANOVA ($F_{2.28}$ = 0.075, p = 0.928)
Locomotor activity	(total number of squares crossed (within 30 sec.)	19.2 (±1.46)	18.6 (±2.28)	17.2 (±3.27)	one way ANOVA ($F_{2.28}$ = 0.413, p = 0.666)
Gait	(0 = fluid movement (3 mm pelvic elevation), 1 = lack of fluid movement (> 3 mm pelvic elevation))	0	0	0	n.s.
Tail elevation	(0 = horizontal extension, 1 = dragging, 2 = elevated/straub tail)	0	0	0	n.s.
Startle response	(0 = none, 1 = preyer reflex, 2 = reaction in addition to preyer reflex)	1.1 (±0.12)	1.12 (±0.15)	1.13 (±0.16)	one way ANOVA ($F_{2.28}$ = 2.149, p = 0.135)
Touch escape	(0 = no response, 1 response to touch, 2 = flees prior to touch)	1 (±0.26)	1 (±0.31)	1.1 (±0.17)	one way ANOVA ($F_{2.28}$ = 0.124, p = 0.884)
Grip strength forepaw		3.45 (±0.23)	3.38 (±0.15)	3.07 (±0.16)	Kurskal Wallis ANOVA (chi-square: 1.216, df = 2, p = 0.526)
Grip strength all paw		6.38 (±0.35)	6.31 (±0.26)	5.78 (±0.26)	one way ANOVA ($F_{2.28}$ = 1.216, p = 0.311)
Positional passivity	(0 = struggles when held by tail, 1 = no struggle)	0	0	0	n.s.
Skin color	(0 = blanched, 1= pink, 2 = birght, deep red)	0.5	0.5	0.5	n.s.
Trunk curl	(0 = present, 1 = absent)	0	0	0	n.s.
Limb grasping	(0 = absent, 1 = present)	0	0	0	n.s.
Pinna refelx	(0 = present, 1 = absent)	0	0	0	n.s.
Corneal reflex	(0 = present, 1 = absent)	0	0	0	n.s.
Contact righting reflex	(0 = present, 1 = absent)	0	0	0	n.s.
Evidence of biting	(0 = none, 1 = biting in response to handling, 2 = excessive activity)	0	0	0	n.s.
Vocalization	(0 = none, 1 = vocal)	0.21 (±0.11)	0.31 (±0.16)	0.24 (±0.13)	one way ANOVA ($F_{2.28}$ = 0.2.72, p = 0.764)
